# Interleukin‐2‐inducible T‐cell kinase (Itk) signaling regulates potent noncanonical regulatory T cells

**DOI:** 10.1002/ctm2.625

**Published:** 2021-12-17

**Authors:** Mahinbanu Mammadli, Rebecca Harris, Liye Suo, Adriana May, Teresa Gentile, Adam T. Waickman, Alaji Bah, Avery August, Elmar Nurmemmedov, Mobin Karimi

**Affiliations:** ^1^ Department of Microbiology and Immunology SUNY Upstate Medical University Syracuse New York USA; ^2^ Department of Pathology SUNY Upstate Medical University Syracuse New York USA; ^3^ Department of Hematology SUNY Upstate Medical University Syracuse New York USA; ^4^ Department of Biochemistry and Molecular Biology SUNY Upstate Medical University Syracuse New York USA; ^5^ Department of Microbiology and Immunology, College of Veterinary Medicine Cornell University Ithaca New York USA; ^6^ Department of Translational Neurosciences Saint John's Cancer Institute Santa Monica California USA

**Keywords:** canonical Tregs, CCR7, CTLA‐4, CXCR3, GVHD, GVL, ICOS, Itk, noncanonical Tregs, PD‐1, SLP76pTYR, Tregs

## Abstract

Regulatory T cells (Tregs) play an important role in controlling autoimmunity and limiting tissue damage and inflammation. IL2‐inducible T cell kinase (Itk) is part of the Tec family of tyrosine kinases and is a critical component of T cell receptor mediated signaling. Here, we showed that either genetic ablation of Itk signaling or inhibition of Itk signaling pathways resulted in increased frequency of “noncanonical” CD4^+^CD25^−^FOXP3^+^ Tregs (ncTregs), as well as of “canonical” CD4^+^CD25^+^FOXP3^+^ Tregs (canTregs). Using in vivo models, we showed that ncTregs can avert the formation of acute graft‐versus‐host disease (GVHD), in part by reducing conventional T cell proliferation, proinflammatory cytokine production, and tissue damage. This reduction in GVHD occurred without disruption of graft‐versus‐leukaemia (GVL) effects. RNA sequencing revealed that a number of effector, cell adhesion, and migration molecules were upregulated in *Itk* ^–/−^ ncTregs. Furthermore, disrupting the SLP76: ITK interaction using a specific peptide inhibitor led to enhanced Treg development in both mouse and primary human cells. This peptide inhibitor also significantly reduced inflammatory cytokine production in primary GVHD patient samples and mouse T cells without causing cell death or apoptosis. We provide evidence that specifically targeting Itk signaling could be a therapeutic strategy to treat autoimmune disorders.

## Graphical abstract

Targeting Itk signaling resulted in increased CD25^+^ FOXP3^+^ canonical and CD25^−^, FoxP3^+^ noncanonical Tregs. Noncanonical Tregs inhibited GVHD induced by conventional CD8^+^ T cells, and reduced production of inflammatory cytokines in mice, without disrupting GVL effects. Disrupting ITK signaling using a specific peptide inhibitor reduced proinflammatory cytokine production by T cells in GVHD patient samples.

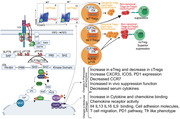

## 1 INTRODUCTION

Regulatory T cells (Tregs) play an important role in immunoregulation and promotion of immunological tolerance.^1^ Tregs have been used as immunotherapy in the bone marrow and solid organ transplantation, autoimmune diseases, and allergy.^2^ In allogeneic hematopoietic stem cell transplantation (allo‐HSCT), Tregs play a significant role in the prevention of graft‐versus‐host disease (GVHD).^3^ However, there are several challenges to the use of Tregs as immunotherapy. One of these challenges is the low number of Tregs in peripheral blood.^4^ Expansion of the Treg population is challenging, as with in vitro expansion these cells lose expression of FOXP3, the master regulatory transcription factor for Tregs.^5^, ^6^ Broad application of the use of Tregs as a therapeutic approach requires standardization of Treg expansion methods and dosing.^7^ Inducible Tregs (iTregs) can be easily generated in vitro, but controversial pre‐clinical findings and phenotype instability have hampered their translation into the clinic.^8^

Several studies have established a direct association between GVHD severity and the number of Tregs present in recipient circulation or tissues post‐transplantation.^9^, ^10^ Although these studies have limitations in establishing the direct role of Treg cells in the severity of GVHD, other studies have demonstrated that depletion of Tregs led to the onset of acute GVHD, while adding Treg cells significantly ameliorated the development of GVHD.^11^ We have shown that T cells deficient in Interleukin‐2‐inducible T cell kinase (Itk) reduce GVHD without affecting GVL function.^12^, ^13^ We have further shown that disruption of SLP76 and Itk signaling using a novel peptide inhibitor leads to significantly decreased GVHD without affecting GVL function.^13^ The absence of Itk results in enhanced development of FOXP3^+^ Tregs, and knockdown of *Itk* led to enhanced expression of FOXP3 in human CD4^+^ T cells, leading to expansion of suppressive Tregs.^13^, ^14^ We have also recently shown that disruption of SLP76 and Itk signaling led to enhanced expansions of Tregs.^12^ In addition, donor CD4^+^ and CD8^+^ T cells from *Itk* ^–/−^ mice clear tumour cells but have significantly delayed development of GVHD.^12^ We hypothesized that this delay in GVHD pathogenesis might be due to enhanced production of Tregs in *Itk^–/–^* mice.

Canonical regulatory T cells (canTregs) are characterized by stable expression of CD25^+^ and FOXP3^+^, while noncanonical regulatory T cells (ncTregs) are FOXP3^+^ cells that do not express CD25.^15^, ^16^ Even though inhibitory mechanisms of regulatory T cells may differ in vitro and in vivo, there are multiple mechanisms that regulatory T cells can use to suppress conventional T cells.^17^ We have recently shown that the T cell transcription factor T Cell Factor‐1 (TCF‐1) regulates production of noncanonical Tregs in a cell‐intrinsic manner.^16^ Here, we have explored the role of Itk in ncTregs, as well as the suppressive ability of ncTregs in GVHD.

We found that both canTregs and ncTregs are significantly enhanced in *Itk* ^–/−^ mice, with cell frequencies increased by 30% and 90%, respectively, compared to C57BL/6 (WT) mice. Furthermore, we found that *Itk* ^–/−^ canTregs and ncTregs more frequently have an effector Treg phenotype compared to WT canTregs and ncTregs, respectively (as defined by CD44^hi^ and CD62L^low^). Furthermore, we also confirmed that central memory CD8^+^ and effector memory CD4^+^ T cell populations are significantly increased compared to in WT mice as shown previously.^18^, ^19^, ^20^ ncTregs from both WT or *Itk* ^−/–^ mice expressed higher levels of PD‐1 compared to canTregs. Expression of CXCR3, which is shown to be an important chemokine receptor for regulatory T cells in preventing GVHD,^21^ was significantly increased in canTregs and ncTregs from *Itk* ^–/−^ mice compared to canTregs and ncTregs from WT mice. We also show that *Itk* ^–/−^ ncTregs express significantly higher levels of CTLA‐4 when cultured with or without anti‐CD3 and anti‐CD28 antibodies, with no differences in the expression levels of IL‐2 and IL‐10, compared to WT canTregs or ncTregs.

Our data further show that *Itk^−/–^* canTregs and ncTregs significantly suppress conventional T cell proliferation in vivo. *Itk^−/–^* canTregs and ncTregs reduced damage in GVHD target organs, and recipient GVHD induced mice treated with *Itk^−/–^* canTregs and ncTregs had reduced proinflammatory cytokine production in serum. RNA sequencing data revealed that in *Itk^–/–^* canTregs, genes involved in the cell cycle were differentially upregulated compared to WT canTregs. We also observed that genes involved in cytokine and chemokine receptor activity, including regulators and effector molecules, were differentially upregulated in *Itk^−/–^* ncTregs compared with *Itk^−/–^* and WT canTregs.

Finally, our data show that primary human or GVHD patient T cells treated with our novel peptide inhibitor, SLP76pTYR,^22^ have significantly reduced the production of inflammatory cytokines without inducing apoptosis or toxicity. Our studies identify the Itk signaling pathway as a potential therapeutic target for modulation of Treg populations, with potential benefits for clinical treatment of T cell‐mediated GVHD. This strategy could also be used to enhance Treg production for the treatment of other autoimmune disorders.

## 2 METHODS AND MATERIALS

### 2.1 Animal used in this manuscript

ITK deficient mice were generated by the August laboratory (Liao and Littman, 1995).

We have generated C57BL/6 WT Luc mice as described by our previous publication (Mahinbanu Mammadli, 2021). B6.SJL‐*Ptprc^a^Pepc^b^*/ and BALB/c H‐2K^d^ background mice were obtained from the Charles River mouse facility, and C57BL/6j H‐2K^b^ background mice were purchased from the Jackson Laboratories. WT C57BL/6‐FOXP3^RFP^ and ITK‐deficient FOXP3^RFP^ mice were provided by the August laboratory, and were previously generated as described before.^23^ 8–12‐week‐old age‐ and sex‐matched mice were used for all experiments. Animal maintenance and experimentation were approved by the Upstate Medical University IACUC committee with IACUC #433.

### 2.2 Chemicals and cell lines were used in this manuscript

All flow cytometry antibodies were obtained from Biolegend. Mouse and human antibodies for cell culture included: mouse anti CD3 (cat#100102) and mouse anti CD28 (cat# 102116) monoclonal antibodies were used for mouse T cell activation, while human anti CD3 OKT3 (cat#317301) and human anti CD28 (cat#302902) monoclonal antibodies were used for human T cell activation. Mouse flow cytometry antibodies were: αCD3 conjugated with BV605, αCD4 conjugated with PE, αCD8 conjugated with PE/Cy7, αCD25 conjugated with BV421, αFOXP3 conjugated with APC, αH‐2K^b^ conjugated with Pacific Blue, αIFN‐γ conjugated with APC, αTNF‐α conjugated with FITC, α CTLA4 conjugated with PE, αIL‐2 conjugated with Pe/Cy7, αIL‐10 conjugated with APC/Cy7, αCD44 conjugated with PerCP, αCD62L conjugated with APC/Cy7, αCXCR3 conjugated with PerCP/Cy5.5, αPD‐1 conjugated with BV785, αICOS conjugated with PE, αCCR7 conjugated with BV605 and αAnnexin conjugated with FITC. Human flow cytometry antibodies were: αCD3 conjugated with APC, αCD4 conjugated with PE, αCD8 conjugated with Pacific Blue, αCD25 conjugated with PerCP/Cy5.5, αFOXP3 conjugated with Pacific Blue, αIFN‐γ conjugated with APC and αTNF‐α conjugated with Pe/Cy7. We used a multiplex ELISA kit from Biolegend (LEGENDplex Mouse Th cytokine panel 12‐plex kit, cat no. 741044) and a customized mouse B cell kit to perform serum cytokine assays. In addition, luciferase substrate was acquired from Gold Bio (St Louis, MO). All *ex vivo* cells were analyzed on a Fortessa machine for flow analysis (from Becton Dickinson Biosciences, BD). All flow cytometry data were analyzed with FlowJo as described.^12^, ^13^, ^22^

CD4^+^ lymphocytes were isolated from the mouse spleen using magnetic beads conjugated to anti‐CD4. We also used column‐based cell separations (from Miltenyi Biotech, Auburn, CA); then cells were stained for surface molecules. Cells were sorted using a BD FACS sorter (Aria IIIu). We obtained over 95% pure sorted cells unless otherwise specified. Reagents used for cell culture and other chemicals were purchased from Sigma‐Aldrich (St. Louis, MO) and Invitrogen (Grand Island, NY), unless stated otherwise. Generation of the primary B‐ALL blast cells was described earlier.^2^, ^3^

### 2.3 Allo‐HSCT and GVL studies

For GVHD experiments, we used an allogeneic MHC‐mismatch mouse model (WT C57Bl/6 H‐2K^b^ was transplanted into BALB/c H‐2K^d^). BALB/c mice were lethally irradiated (800 cGy divided into two doses of 400 cGy). Recipient animals were transplanted via IV injection with 10 × 10^6^ bone marrow cells, with T cells being depleted from bone marrow by CD90.2 beads. Animals were also transplanted with CD4^+^ T cells and CD8^+^ T cells mixed at a 1:1 ratio from WT or ITK^–/–^ mice, and these ex vivo cells were purified using column‐based cell separations. In addition, B‐ALL primary blast cells were transduced with lentiviral particles carrying firefly luciferase as described (B‐ALL luc).^12^, ^13^, ^22^ Animals were challenged with 2×10^5^ B‐ALL luc cells as a readout for GVL studies.^12^, ^13^, ^22^ Recipient mice were checked for bioluminescence using bioluminescent imaging (BLI) and for clinical symptoms of GVHD and weight loss for 50 days.

### 2.4 Allo‐HSCT and GVL studies with Treg treatment

For experiments on preventing GVHD with Tregs, host BALB/c H‐2K^d^ animals were lethally irradiated and transplanted with 10 × 10^6^ bone marrow cells, which had been depleted of T cells by negative selection using CD90.2 beads. 1 × 10^6^ WT CD8^+^ T cells and 1 × 10^5^ primary tumour cells (B‐ALL) were administered intravenously. Tregs were FACS sorted from WT mice or *Itk* ^–/−^ mice using FOXP3 IRES RFP (FOXP3^RFP^) and expression of CD4 and CD25. Recipient mice were treated with 0.5 × 10^6^ noncanonical or canonical Tregs from *Itk ^–/–^* mice, canonical Tregs from WT mice or left untreated, and were evaluated for clinical symptoms of GVHD and weight loss for more than 40 days. To determine the clinical score of GVHD, recipient mice were examined two to three times per week as described.^6^ When recipient animals lost more than 30% of their original body weight, they were euthanized. Twice a week after tumour injection, IVIS 200 Imaging System (Xenogen) was used to evaluate tumour growth by bioluminescence as described earlier.^12^, ^13^, ^22^

### 2.5 Cytokine production assay

On day 7 post‐transplantation (with BM, WT CD8^+^ T cells, and FACS sorted canonical or noncanonical Tregs from *Itk^–/–^* mice or canonical Tregs from WT mice), lymphocytes were isolated from the secondary lymphoid organs (spleen and inguinal lymph nodes) of recipients. Cells were either in vitro activated with anti CD3 and anti CD28 or left unstimulated, and cultured for a total of 6 h with GolgiPlug (BD Cytofix/Cytoperm Plus kit cat#555028). These *ex vivo* cells were stained for H‐2K^b^ to identify donor cells (C57Bl/6 cells), CD4, CD3 and CD8. Cells were then stained for cytokines against IFN‐γ conjugated with APC and against TNF‐α conjugated with FITC intracellularly.

### 2.6 Cytokine Serum ELISA

Host BALB/c animals were irradiated lethally with 800 cGy total, divided into two doses of 400 cGy. Animals were then transplanted with 10 × 10^6^ bone marrow cells, which were depleted of mature T cells by negative selection using CD90.2 beads. Animals were also transplanted with 1 × 10^6^ MACS‐sorted WT CD8^+^ T cells administered intravenously. In addition, transplanted mice were given 0.5 × 10^6^ noncanonical or canonical Itk‐deficient Tregs or canonical WT Tregs or left untreated. On day 7 after transplantation, we euthanized mice, total blood was collected, and serum was separated by centrifugation. The serum was examined for numerous cytokines with a Mouse Th Cytokine LEGENDplex ELISA assay (Biolegend cat no 741044), following kit instructions. IL‐2, TGF‐β and IL‐10 were also tested using a customized Mouse B Cell Panel LEGENDplex ELISA assay (Biolegend, custom order). A BD LSRFortessa cytometer was used to collect the data, and collected data were analyzed by utilizing LEGENDplex software (provided with kit via Biolegend).

### 2.7 Cell culture for different regulatory T cell markers

We obtained total splenocytes from WT or *Itk^–/–^* mice to test FOXP3, CD25, CTLA‐4 and IL‐10 markers in regulatory T cells. Cells were cultured in LAK media with GolgiPlug from the Cytofix/Cytoperm Plus kit (cat#555028 from BD) for 6 h. *Ex vivo* cells were cultured and stimulated with anti‐CD3 (1 μg/mL) and anti‐CD28 (2 μg/mL) for 6 h, or cultured but not stimulated. These *ex vivo* cells were stained with FOXP3, CD25, CTLA‐4 and IL‐10 markers, and data were collected on the Fortessa cytometer.

### 2.8 Cell death assay

To determine the level of apoptosis and cell death after SLP76pTYR treatment, healthy human PBMCs and mouse splenocytes were isolated, cultured at 3×10^6^ cells per ml, and treated with 1 μg of SLP76pTYR or vehicle for 1 h. These *ex vivo* cells were stained for the surface markers against CD3, against CD8 and against CD4. These cells were also stained with Annexin V‐FITC (V13242 life) and LIVE/DEAD Near‐IR (L34976 life). To identify cell subsets, we gated on live cells, NIR^+^ Annexin V^+^ for dead cells and Annexin V^+^ for apoptotic cells.

### 2.9 RNA sequencing

C57BL/6‐FOXP3^RFP^ and *Itk^–/–^‐*FOXP3^RFP^ mice were euthanized; CD4^+^ T cells were MACS purified; and CD3, CD4, CD25 and FOXP3 markers were used to determine canonical and noncanonical Tregs. Using a FACS Aria IIIu cell sorter (BD Biosciences), CD3^+^ CD4^+^ CD25^+^ FOXP3^+^ (canonical) Tregs and CD3^+^ CD4^+^ CD25^–^ FOXP3^+^ (noncanonical) Tregs were sorted into Trizol. Only CD3^+^ CD4^+^ CD25^+^ FOXP3^+^ (canonical) Tregs could be sorted from C57BL/6‐FOXP3^RFP^ CD4^+^ cells because the noncanonical C57BL/6‐FOXP3^RFP^ Tregs percentage was so small that we could not sort enough cells for downstream applications. RNA extraction and library prep of sorted Tregs were performed. RNA library preps were sent to the University at Buffalo Genomics Core facility for RNA sequencing and analysis using an Illumina NovaSeq 6000 sequencer.

Initially, the first six samples (two per group) were library prepped using the Bio‐Rad SEQuoia library prep kit, and the last three samples (one sample for each group) were library prepped using the SMART‐Seq HT kit (Takara Bio). These nine samples were all sequenced in the same run. Still, the six samples (two samples in each group) library prepped using the SEQuioa kit resulted in an insufficient number of reads, making a comparison between groups impossible. Therefore, we had to re‐prep and sequence the first six samples again using the SMART‐seq HT kit. The batch effect from sequencing runs between the six samples re‐prepped and the three original samples were later removed. We created three groups, WT canonical, *Itk^–/–^* canonical, and *Itk^–/–^* noncanonical Tregs, to determine the differences between groups of Tregs.

For RNA sequencing analysis, we used R language programming (Version 4.0.4), as well as RStudio interface (Version 1.4.1106), and Bioconductor to process and analyze the data. Pseudoalignment was performed using Kallisto (version 0.46.2)^24^ to determine the transcript abundance of samples. We normalized the transcript per million (TPM) values. We applied the linear model by using the empirical Bayes method and the Voom and Limma R packages^25^ to remove the batch effect. We used Bonferroni and Hochberg^26^ adjusted *p* value and (FDR) of ≤ .05 or FDR ≤ .1 to describe the differential genes expression. We used the G:Profiler toolset and g:GOSt function for GO annotation analysis.

We used C2 and C7 pathways collections (MSigDB) for the identification of important pathways and to perform Gene Set Enrichment Analysis (GSEA). RNA sequencing analysis data uploaded at https://www.ncbi.nlm.nih.gov/geo/ with accession number GSE185327.

### 2.10 In vivo T cell proliferation assay

Host BALB/c animals were irradiated lethally with 800 cGy total, divided into two doses of 400 cGy. These mice were then transplanted with 10 × 10^6^ bone marrow cells with T cells being depleted as above. Animals were also transplanted with 1 × 10^6^ WT *luc* ^+^ CD8^+^ T cells, and 0.5 × 10^6^ FACS sorted canonical or noncanonical *Itk^–/–^* Tregs or canonical Tregs from WT mice, which were administered intravenously to detect in vivo T cell proliferation through bioluminescence imaging (BLI). IVIS‐50 was utilized to image recipient BALB/c mice every day for 7 days. Bioluminescence was quantified as described.^12^, ^13^, ^22^ One‐way ANOVA was performed for statistical analysis followed by Tukey's multiple comparison test.

### 2.11 Post‐transplant Treg phenotype assay

To detect the canTregs and ncTregs post‐transplant, BALB/c animals were irradiated and then transplanted with 10 × 10^6^ bone marrow cells without mature T cells. Animals were also transplanted with 1 × 10^6^ WT CD8^+^ T cells, and 1 × 10^6^ FACS sorted CD4^+^ FOXP3‐RFP^+^ total Tregs (CD25^+^ and CD25^‐^) from *Itk^–/–^*, or WT mice, which were transplanted intravenously. At 7 days post‐transplantation, splenocytes and liver cells were removed and donor cells were isolated. Donor cells were stained by H‐2K^b^, and we also stained against CD3, against CD4, against CD25 and against FOXP3 markers.

### 2.12 Histopathological examination

Animals were transplanted with 10 × 10^6^ bone marrow cells without T cells and were also transplanted with 1 × 10^6^ CD8^+^ T cells from WT C57BL/6 mice and 0.5 × 10^6^ FACS sorted canonical or noncanonical Tregs from *Itk^–/–^* mice or canonical Tregs from WT mice, which were administered intravenously in lethally irradiated mice. On day 7 post‐transplantation, we isolated liver and small intestines, which were obtained from host mice, and stained them with H&E. A pathologist graded the H&E slides blindly, and photos were also taken of the sectioned organs. The Chi‐square test and Kruskal Wallis test followed by Dunn's multiple comparison test were conducted to evaluate the statistical difference between groups.

### 2.13 Human patient samples

T cells were purified from human PBMCs of either healthy donor or GVHD patient samples, using isolation methods we described earlier (Karimi et al., 2005) using Ficoll–Paque density centrifugation. Tregs were cultured at 3 × 10^6^  cells per ml with 10% RPMI media and activated with anti‐CD3 (2.5 μg/ml) along with 5 μg/ml Polybrene (Millipore Sigma cat#TR‐1003‐G) and 1 μg of SLP76pTYR or vehicle. The cells were plated in a 12‐well plate for 5 to 24 h in an incubator (37°C, 7% CO_2_). After incubation, the cells were stained for Treg markers. For cytokine expression experiments, the 3 × 10^6^ cells/ml were resuspended in RPMI media and stimulated with anti‐CD3 (2.5 μg/ml) and anti‐CD28 (2.5 μg/ml), along with 5 μg/ml Polybrene, 1 μl/ml GolgiPlug as described and 1 μg of SLP76pTYR or vehicle. Cells were plated in a 12‐well plate for 6 h and incubated (37°C, 7% CO_2_). After incubation, the cells were stained for extracellular markers and fixed using the BD Cytofix/Cytoperm Plus kit. Twenty‐four hours later, the cultured cells were permeabilized and were stained for TNF‐α and IFN‐γ intracellularly and analyzed by flow cytometry.

### 2.14 Cellular thermal shift assay (CETSA)

Purified mouse T cells were cultured in a 10% RPMI medium with 10% FBS as described earlier (Karimi et al., 2005; Karimi et al., 2014). To find out the initial melting profile of ITK, fresh T cell lysate was added in non‐denaturing buffer and transferred in a 96‐well PCR plate in the above medium (approx. 10 000 cells/well/50 μl) concentration. T cell lysates were then exposed to a temperature gradient (37–60°C) for 20 min. Then, lysates were centrifuged at 14 000 RPM to remove the unstable protein content. Subsequently, the supernatant was run on SDS‐PAGE gel, and immuno‐detection was conducted for ITK using the corresponding primary antibody. LI‐COR C‐Digit Blot Scanner was utilized to detect and quantify the band intensity, and the *T*_agg_ (50) and *T*_agg_ (75) values were determined for ITK. In the next run, fresh lysates from purified mouse T cells were treated at different concentrations with threefold dilutions (20, 6.6, 2.2, 0.75, 0.25, 0.08 and 0.027 μM) of the peptide SLP76pTYR or the DMSO control. Then samples were exposed to heat challenge at *T*_agg_ (50) for 20 min, followed by centrifugation to remove the unstable protein. After immuno‐blotting the samples, bands of remaining stable ITK were measured and normalized to the loading control, and using GraphPad Prism software, were plotted. EC50 values of engagement for both compounds with ITK were subsequently calculated.

### 2.15 Statistical analyses

Depending on the dataset, we used GraphPad Prism version 9 for statistical and quantitative analysis, using either one‐way ANOVA, 2‐way ANOVA or Student's *t*‐test. Tukey's multiple comparisons tests followed all the ANOVA analyses. Kruskal Wallis test was used for histology grade analyses and was followed by Dunn's multiple comparison test. Statistics are represented as means with standard deviation. Unless otherwise mentioned, according to power analyses, all experiments were performed with a minimum of three mice per group and repeated several times. We used Kaplan–Meier survival analyses for survival experiments. Unless otherwise mentioned, all tests were confirmed by two‐sided tests, and the *p* values equal or less than .05 were considered to be significant. A minimum of three to five mice per group was used for transplant experiments, with at least two repeats. Unless otherwise noted, all in vitro experiments were conducted a minimum of three times with one to three replicates per condition per experiment. Three replicates per group and per condition were used to conduct the RNAseq analysis.

## 3 RESULTS

### 3.1 *Itk^−/–^* T cells delay GVHD even at high numbers

We utilized MHC‐mismatched donors and recipients, with T cell‐depleted bone marrow (_TCD_BM) from C57Bl/6 (B6, WT) mice, donor T cells from B6 WT or *Itk* ^–/−^ mice on a C57BL/6 background (MHC haplotype of b) and lethally irradiated BALB/c (MHC haplotype of d) mice as recipients, in order to cause GVHD. For GVL experiments, B‐ALL primary blasts^23^ (B‐ALL‐*luc*) were used as described previously.^12^, ^13^ Using this model, we recently reported that 1 × 10^6^ to 2 × 10^6^ *Itk*‐deficient CD4^+^ and CD8^+^ T cells do not cause GVHD, compared to the same number of CD4^+^ or CD8^+^ T cells from WT mice which cause acute GVHD.^12^ Here, we determined the maximum number of CD4^+^ and CD8^+^ T cells from WT that would clear the tumour without inducing GVHD.

**FIGURE 1:**
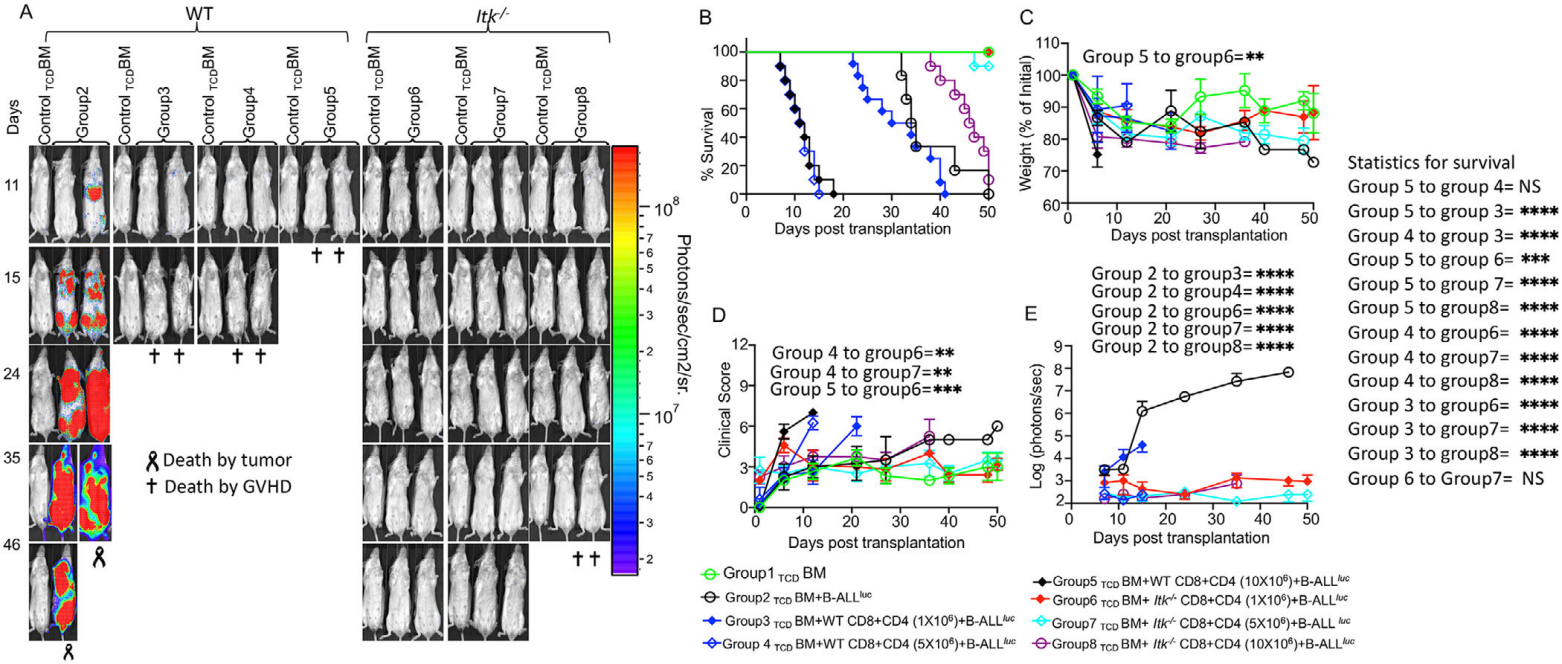
T cells from *Itk^−/–^* mice delay GVHD even at high doses. (A) We have used MHC‐mismatched donors and recipients in order to induce GVHD, T cell‐depleted bone marrow (_TCD_BM) from C57Bl/6 (B6) mice, donor T cells from C57BL/6 (B6) WT or *Itk* ^–/−^ C57BL/6 background mice (MHC haplotype b) were administered in the lethally irradiated BALB/c (MHC haplotype d) recipients. Different numbers of CD4^+^ and CD8^+^ T cells from WT or *Itk^–/–^* mice were purified and mixed at a 1:1 ratio and transplanted into lethally irradiated BALB/c mice, along with 2 × 10^5^ B‐ALL‐*luc* cells and 10 × 10^6^ T cell depleted bone marrow cells. *Group 1* received 10 × 10^6^ T cell depleted bone marrow only (labelled as _TCD_BM). *Group 2* received 10 × 10^6^ T cell depleted bone marrow along with 2 × 10^5^ B‐ALL‐*luc* cells (_TCD_BM+B‐ALL‐*luc*). *Group 3* was transplanted with 10 × 10^6^ T cell depleted bone marrow with 0.5 × 10^6^ purified CD8^+^ and 0.5 × 10^6^ CD4^+^ T cells from WT C57Bl/6 mice (1:1 ratio), along with 2 × 10^5^ B‐ALL‐*luc*+ cells (_TCD_ BM+WT CD8+CD4 (1 × 10^6^) + B‐ALL‐*luc*). *Group 4* received 10 × 10^6^_TCD_BM with 2.5 × 10^6^ purified CD8^+^ and 2.5 × 10^6^ CD4^+^ T cells from WT C57Bl/6 mice (1:1 ratio), along with 2 × 10^5^ B‐ALL‐*luc*+ cells (_TCD_BM+WT CD8+CD4 (5 × 10^6^) +B‐ALL‐*luc*). Group 5 received 10 × 10^6^_TCD_BM with 5 × 10^6^ purified WT CD8^+^ and 5 × 10^6^ CD4^+^ T cells from WT C57Bl/6 mice (1:1 ratio), along with 2 × 10^5^ B‐ALL‐*luc*+ cells (BM+WT CD8+CD4 (10 × 10^6^) +B‐ALL‐*luc*). *Group 6* was transplanted with 10 × 10^6^_TCD_BM with 0.5 × 10^6^ purified *Itk^–/–^* CD8^+^ and 0.5 × 10^6^ CD4^+^ T cells (1:1 ratio), along with 2 × 10^5^ B‐ALL‐*luc*+ cells (_TCD_ BM+ *Itk^–/–^* CD8+CD4 (1 × 10^6^) +B‐ALL‐*luc*). *Group 7* was transplanted with 10 × 10^6^_TCD_BM with 2.5 × 10^6^ purified *Itk^–/–^* CD8^+^ and 2.5 × 10^6^ CD4^+^ T cells (1:1 ratio), along with 2 × 10^5^ B‐ALL‐*luc*+ cells (_TCD_ BM+ *Itk^–/–^* CD8+CD4 (5 × 10^6^) +B‐ALL‐*luc*). *Group 8* was transplanted with 10 × 10^6^_TCD_BM with 5 × 10^6^ purified *Itk^–/–^* CD8^+^ and 5 × 10^6^ CD4^+^ T cells (1:1 ratio), along with 2 × 10^5^ B‐ALL‐*luc*+ cells (_TCD_ BM+ *Itk^–/–^* CD8+CD4 (10 × 10^6^) +B‐ALL‐*luc*). Recipient BALB/c mice were imaged using IVIS 50 three times a week. The mice were monitored for survival (B), changes in body weight (C), and clinical score (D) for about 50 days post BMT. (E) Quantitated luciferase bioluminescence of tumour growth. Statistical analysis of differences in survival (B) for different groups of recipient BALB/c mice is shown on the right. Statistics for differences in weight loss (C), score (D), and bioluminescence (E) are shown within the respective graphs. Groups of recipient BALB/c transplanted with T cells from WT mice were compared among each other and compared to recipient BALB/c transplanted with T cells from *Itk^–/–^* mice. Statistical analysis for survival and the clinical score was performed using a log‐rank test and one‐way ANOVA with Tukey's test, respectively. For weight changes and clinical score, one representative of two independent experiments is shown (*n* = 3 mice/group for BM alone; *n* = 5 experimental mice/group for all 7 other groups). Survival is a combination of two experiments. Symbol meaning for *p* values are: ns—*p* > .05; *p ≤ .05; **p ≤ .01; ***p ≤ .001; ****p ≤ .0001. Note: Control mouse is a recipient mouse given _TCD_BM only (group 1), used as a negative control for BLI (no bioluminescent tumour cells were given)

Recipient BALB/c mice transplanted with 10 × 10^6^ T cell‐depleted bone marrow cells (_TCD_BM cells) from wild‐type WT C57BL/6 mice as a bone marrow only control (group 1) all survived for more than 50 days post allogeneic transplantation with no signs of GVHD (Figure 1A–D). The second cohort of recipient BALB/c mice given 10 × 10^6^  T cell‐depleted bone marrow cells and also given 2 × 10^5^ B‐ALL‐luc cells (group 2) all developed tumours as measured by bioluminescence (Figure 1A,E). Furthermore, all of these animals had to be euthanized as a result of tumour burden (Figure 1A). Another cohort of recipient BALB/c mice was given 10 × 10^6^_TCD_BM cells along with 0.5 × 10^6^ CD4^+^ and 0.5 × 10^6^ CD8^+^ T cells from WT C57BL/6 mice, and 2 × 10^5^ B‐ALL‐luc cells (group 3). This group of recipient mice was able to clear the tumour cells but developed acute GVHD after 2 weeks (Figure 1A–D). The fourth cohort of recipient BALB/c mice was transplanted with 10 × 10^6^ 1 bone marrow cells and 2.5 × 10^6^ CD4^+^ and 2.5 × 10^6^ CD8^+^ T cells from WT C57BL/6 mice, and also given 2 × 10^5^ B‐ALL‐ luc cells (group 4). These animals were also able to clear tumour cells but developed acute GVHD, and all recipient animals had to be euthanized due to GVHD within 2 weeks (Figure 1A–D). The fifth cohort of recipient BALB/c mice was given 10 × 10^6^ 1 _TCD_BM cells along with 5 × 10^6^ CD4^+^ and 5 × 10^6^ CD8^+^ T cells from WT C57BL/6 mice, and these recipient mice were challenged with 2 × 10^5^ B‐ALL‐luc cells (group 5). All transplanted mice were able to clear transplanted cancer cells, but all animals developed acute GVHD within 20 days (Figure 1A–D).

In contrast, the sixth cohort of recipient BALB/c mice was transplanted with 10 × 10^6^ bone marrow cells, 0.5 × 10^6^ CD8^+^ T cells and 0.5 × 10^6^ CD4^+^ from ITK‐deficient mice in B6 background, and 2 × 10^5^ B‐ALL‐luc cells (group 6). These mice were able to clear luciferase expressing cancer cells and did not develop acute GVHD, with all animals surviving more than 50 days with minimal GVHD scores (Figure 1A–D). The seventh group of recipient BALB/c mice was transplanted with 10 × 10^6^ 1bone marrow cells, and recipient mice were also given 2.5 × 10^6^ CD4^+^ and 2.5 × 10^6^ CD8^+^ T cells from *Itk* ^–/−^ mice and challenged with 2 × 10^5^ luciferase‐expressing B‐ALL tumour cells (group 7). All animals in group 7 cleared the cancer cells, and only 2 of 10 animals developed acute GVHD, with 8 mice surviving to 50 days post transplantation (Figure 1A–D). The final (group 8) cohort of recipient BALB/c mice were transplanted with 10 × 10^6^ bone marrow cells, and given 5 × 10^6^ CD4^+^ mixed with 5 × 10^6^ CD8^+^ T cells from ITK deficient mice. These mice were further challenged with luciferase 2 × 10^5^B‐ALL cells, as for other groups. All transplanted mice were able to clear luciferase expressing cancer cells very quickly, but the animals started to develop GVHD at 40 days post‐transplantation (Figure 1A–D).

Each group of animals as described above was examined for survival (Figure 1B), and monitored for weight changes (Figure 1C). Each animal from all groups was examined three times a week for GVHD clinical score. The clinical scoring included changes in weight, changes in activities and changes in posture. We also examined these mice for fur texture and skin integrity as described in a standard clinical score protocol^27^ (Figure 1D). Cancer cell proliferation as tumour growth was determined by quantification of luciferase bioluminescence of each animal in each group (Figure 1E). Our findings demonstrated that recipient mice transplanted with as high as 10 × 10^6^ donor T cells (both CD4^+^ and CD8^+^ T cells) from mice lacking ITK had delayed induction of GVHD. This was compared with mice transplanted with 1 × 10^6^ CD8^+^ and CD4^+^ T cells from WT mice, where GVHD was rapidly induced. Statistical differences among recipient groups that were transplanted with various numbers of allogeneic cells as described above showed that there are no significant differences between transplanting 5 × 10^6^ and 10 × 10^6^ WT donor T cells (both groups of recipient mice died from severe GVHD) or between transplanting 5 × 10^6^ and 1 × 10^6^ *Itk^–/–^* donor T cells (recipient mice from both groups survived). However, there were significant differences comparing all other groups between each other. The statistically significant increase in survival of the group given 10 × 10^6^ *Itk^–/–^* donor T cells compared to the group given 1 × 10^6^ WT donor T cells suggests that the increase in survival from GVHD is preserved even when transplanting the maximum number of T cells from mice lacking ITK compared with T cells from WT mice (Figure 1B).

### 3.2 ITK suppresses the development of the noncanonical Treg phenotype

We observed that cells from donor mice lacking ITK can delay the development of GVHD, even with higher numbers of donor T cells. Published data have shown that ITK deficiency enhances regulatory T cells (CD25^+^, and FOXP3^+^ cells).^14^, ^22^, ^28^ We hypothesized that these mice may have received higher numbers of these FOXP3^+^ Treg populations, helping to suppress GVHD development. Published data have suggested that Itk is an important component of TCR proximal signal transduction pathways in regulatory Tregs.^14^, ^28^ In addition, we have recently demonstrated that targeting SLP76:ITK interaction may increase regulatory T cells (Tregs)^13^ indicating that ITK negatively regulates Tregs.

**FIGURE 2:**
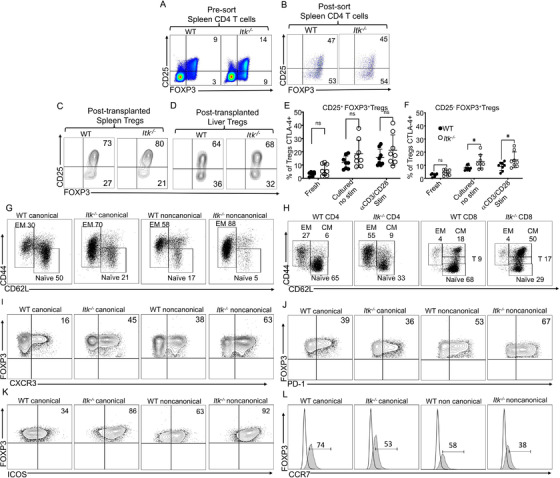
Itk deficiency enhances noncanonical Treg production. (A) Pre‐sorted WT and *Itk^–/–^* CD4^+^ T cells from spleens of naive mice were examined for expression of CD25 and FOXP3 by flow cytometry. (B) Next, for FACS, purified canonical and noncanonical Tregs from WT C57Bl/6 and Itk^–/–^ mice were gated on CD4 and FOXP3 positive T cells and the non‐Treg CD4^+^ T cells were excluded; then Tregs were gated on CD25 and FOXP3 to confirm the canonical and noncanonical Tregs. (C) FACS purified CD4^+^ FOXP3^+^ Tregs from either C57BL/6‐FOXP3^RFP^ or *Itk* ^–/−^ FOXP3^RFP^ mice were transplanted into lethally irradiated BALB/c animals along with CD8^+^ T cells from WT C57Bl/6 mice. At day 7 post‐transplant, donor Tregs from the recipient spleen (C) or liver (D) were gated on H‐2k^b^ and CD3 to identify the donor T cells. Next, T cells were gated on CD4 and CD8 markers, followed by CD4 versus FOXP3 gating to plot CD25 and FOXP3 for determining canonical and noncanonical Tregs. E‐F) CD4^+^ T cells were obtained from naive WT C57Bl/6 mice and *Itk ^–/–^* mice, and either stained immediately or cultured for 6 h with or without anti‐CD3/anti‐CD28. Cells were then stained for CTLA‐4, CD3, CD4, CD25 and FOXP3. CTLA‐4 expression in (E) CD25^+^ FOXP3^+^ (canonical) Tregs from WT or *Itk ^–/–^* mice and (H) in CD25^–^ FOXP3^+^ (noncanonical) Tregs from WT or *Itk ^–/–^* mice. (G) Canonical and noncanonical Tregs from naive WT C57Bl/6 mice and *Itk ^–/–^* mice were examined for CD44 and CD62L expression. (H) Conventional CD8^+^ and CD4^+^ T cells from naive WT C57Bl/6 mice and *Itk ^–/–^* mice were examined for CD44 and CD62L expression. (I) CXCR3 expression in canonical and noncanonical WT or *Itk ^–/–^* Tregs from naive mice. (J) PD‐1 expression in canonical and noncanonical WT or *Itk ^–/–^* Tregs from naive mice. (K) ICOS expression in canonical and noncanonical WT or *Itk ^–/–^* Tregs from naive mice. (L) CCR7 expression in canonical and noncanonical WT or *Itk ^–/–^* Tregs from naive mice. One experiment is shown as a representative from two independent experiments, for statistics data from two to three independent experiments pooled. Statistical analysis was performed using one‐way ANOVA with Tukey's test, *p* value presented with each figure. Symbol meaning for *p* values are: ns, *p* > .05; * *p* ≤ .05; ** *p* ≤ .01; *** *p* ≤ .001; **** *p* ≤ .0001

Our data showed that the loss of ITK resulted in significantly increased canonical regulatory T cells, which express CD25 and FOXP3 (canTregs), and noncanonical regulatory T cells, which are FOXP3^+^ cells that do not express CD25 (ncTregs) (Figure 2A; Figure S1A). These ncTregs are functional cells and have the suppressive ability in some contexts.^15^ Delacher et al. (2020) demonstrated that mice are lacking TCF‐1 exhibit an increased frequency of FOXP3 expression in all T cells, encompassing T cells that normally do not express FOXP3 like CD8^+^T cells.^29^ It was possible that the same effect was occurring in *Itk^–/–^* mice, with FOXP3 simply being expressed more highly in all T cells. Therefore, we examined whether ITK deficiency might also cause aberrant expression of FOXP3. Therefore, we examined the expression of FOXP3 in CD8^+^ T cells (where it would not normally be expressed). Briefly, naive WT and *Itk* ^–/−^ T cells were either stained immediately or cultured for 6 h in the presence of GolgiPlug, and cells were cultured with media as a non‐stimulated group or anti‐CD3/anti‐CD28 (αCD3/CD28 stim. group). We found that the frequency of CD8+ FOXP3^+^ cells (either CD25^+^ or CD25^–^) was not increased among T cells from ITK‐deficient mice. Our data showed that mice lacking ITK do not have aberrant FOXP3 expression on CD8^+^T cells (Figure S1B,C).

Next, we examined whether the canonical and noncanonical Treg populations were maintained post‐transplantation. CD4^+^ T cells were purified using MACS purification from the spleen of either WT or ITK‐deficient mice. These MACS‐purified Treg cells were further purified using FACS sorting using CD25 and FOXP3 markers. The sorted cells were stained for CD25 and FOXP3 by RFP^23^ (Figure 2B). Next, we performed allogeneic transplant experiments as described above, where we used an MHC‐mismatch mouse model of allogeneic transplantation (WT C57Bl/6 into BALB/c, H‐2K^b ^→ mice into H‐2K^d^). The transplanted mice were further treated with sort‐purified Tregs (canonical and noncanonical Tregs). Briefly, recipient BALB/c mice were lethally irradiated as described and transplanted with 10 × 10^6^ T cell‐depleted bone marrow cells (_TCD_ BM). The recipient mice were also transplanted with 1 × 10^6^ CD8^+^ T cells from WT mice and treated with 1 × 10^6^ canonical and noncanonical FACS‐sorted Tregs from either WT C57Bl/6 or ITK‐deficient mice. We have sort‐purified these with CD4^+^, CD25^+^ and FOXP3 with RFP marker (FOXP3^RFP^)^30^ (Figure S2A‐B). Recipient mice were euthanized at day 7 post transplantation. Recipient mouse spleens and livers were examined for donor cells by H‐2K^b^, CD4, CD25 and FOXP3^RFP^.

We observed that both the canonical and noncanonical donor Treg cell populations were maintained in recipient's spleen (Figure 2C; Figure S2C‐D). Although we did not see an increase in ncTregs in recipient mice given *Itk^–/–^* Tregs compared to WT Tregs (as we saw in naive mice), the canTregs from *Itk^–/–^* were still increased in spleen post‐transplantation compared to WT canTregs (Figure 2C; Figure S2C‐D, S2G). We also observed that transplanted Itk^–/–^ncTregs were decreased in the spleen of recipient mice compared to the pre‐sorted Itk^‐/‐^ ncTregs (Figure S2A,C‐D,G). This might be due to these cells migrating from the spleen to GVHD target organs, as a high percentage of them express CXCR3. We also looked at the liver, one of the GVHD target organs, and we observed a slight increase in *Itk^–/–^* canTregs and ncTregs compared to WT Tregs in recipient mice (Figure 2D; Figure S2E–G). We also observed that the small percentages of T cells that were not Tregs from the sorting expanded exponentially following allogeneic transplantation (Figure S2B–F). Altogether, these data suggest that the noncanonical Treg population that was increased in *Itk^–/–^* mice is not a result of activation of canonical Tregs; instead, these noncanonical Tregs are a distinct population and maintain their phenotype after transplantation.

The underlying mechanisms of Treg function are still not clear. However, several studies have indicated that several key molecules such as the use of membrane bound TGF‐β,^30^ expression of FAS and granzyme B,^31^ LAG‐3^32^ or CTLA‐4^33^ may play a role. The other possible mechanisms of Tregs include secretion of inhibitory molecules such as IL10, TGF‐β^34^ or IL‐35;^35^ local competition for growth factors such as consumption of IL‐2^36^ or cytokine deprivation‐induced apoptosis via BCL‐2.^37^ Since CTLA‐4 is an important factor for Treg function and identity,^38^ we examined whether *Itk* ^–/−^ Tregs expressed CTLA‐4, as well as whether they expressed the cytokines IL‐2 and IL‐10.

Our data show that canTregs from ITK‐deficient mice seem to have increased expression of CTLA‐4 (Figure 2E). Among ncTregs, there was no significant difference in proportion that expresses CTLA‐4 between fresh WT and *Itk^–/–^* cells. When we cultured T cells with or without anti‐CD3 and anti‐CD28 antibodies, we observed a significant enhancement of CTLA‐4 expression in ncTregs from ITK‐deficient mice compared to CTLA‐4 expression ncTregs from WT mice (Figure 2F). We also found that there was no difference in expression of IL‐2 and IL‐10 between *Itk* ^–/−^ and WT cells for both canonical and noncanonical Tregs, regardless of culture conditions (Figure S3A–D).

**FIGURE 3:**
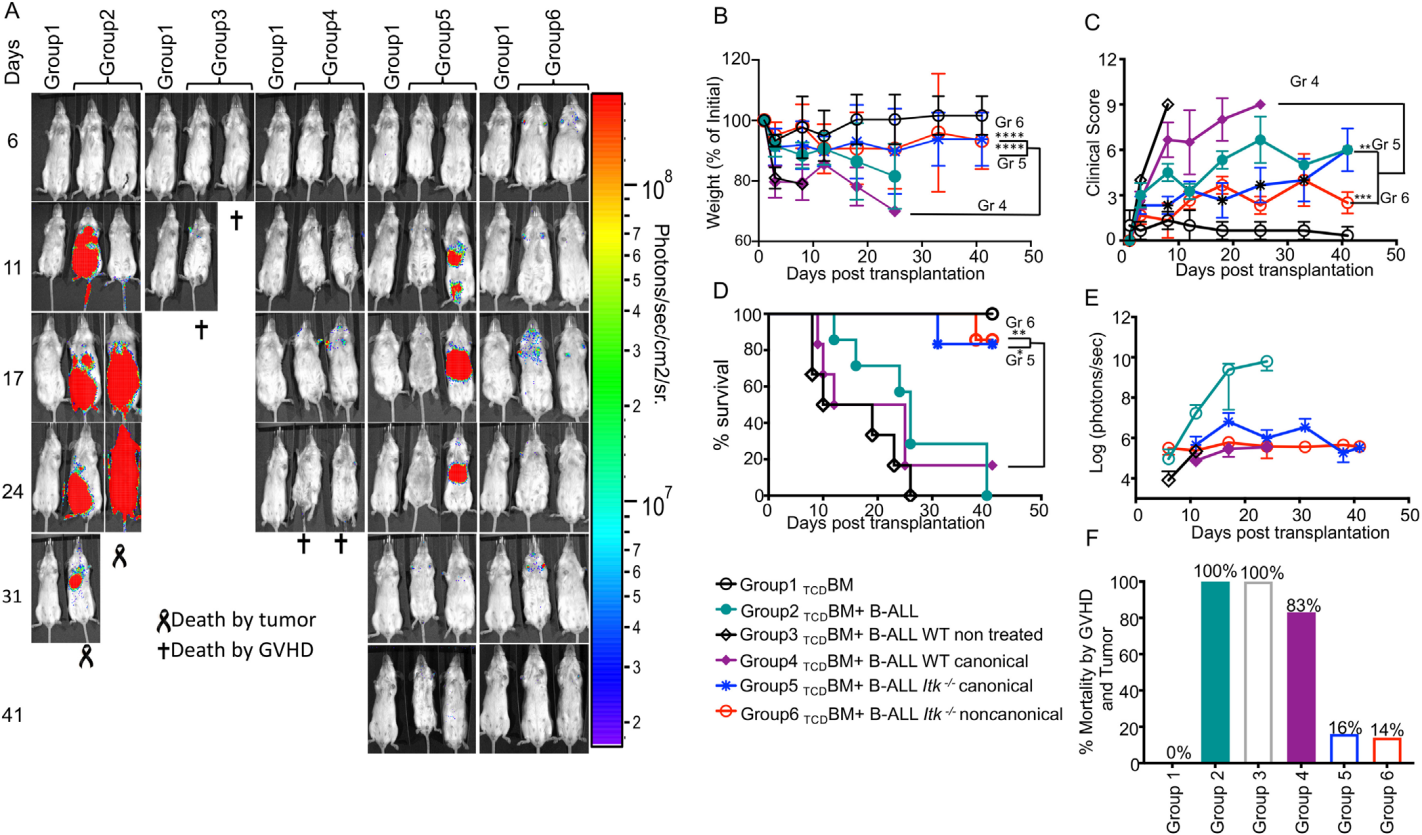
Noncanonical *Itk* ^–/−^ Tregs suppress GVHD but maintain GVL effects. (A) *Group 1* BALB/c recipient mice were lethally irradiated and transplanted with 10 × 10^6^_TCD_BM, alone. *Group 2* BALB/c mice were transplanted with 10 × 10^6^_TCD_BM and 1 × 10^5^ primary tumour cells (B‐ALL‐*luc* ^+^). *Group 3* BALB/c mice were transplanted with 10 × 10^6^_TCD_BM +1 × 10^6^ WT CD8^+^ T cells and 1 × 10^5^ primary tumour cells (B‐ALL‐*luc* ^+^). *Group 4* BALB/c mice were transplanted with 10 × 10^6^_TCD_BM, 1 × 10^6^ WT CD8^+^T cells, and 1 × 10^5^ primary tumour cells (B‐ALL‐*luc* ^+^), and were treated with 0.5 × 10^6^ canonical Tregs from WT C57Bl/6 mice. *Group 5* BALB/c mice were transplanted with 10 × 10^6^_TCD_BM,1 × 10^6^ WT CD8^+^T cells, and1 × 10^5^ primary tumour cells (B‐ALL‐*luc* ^+^), and were treated with 0.5 × 10^6^ canonical Tregs from *Itk^–/–^* mice. *Group 6* BALB/c mice were transplanted with 10 × 10^6^_TCD_BM, 1 × 10^6^ WT CD8^+^T cells, and 1 × 10^5^ primary tumour cells (B‐ALL‐*luc* ^+^), and were treated with 0.5 × 10^6^ noncanonical Tregs from *Itk^–/–^* mice. Tregs were sorted from either WT mice or *Itk* ^–/−^ mice using CD4, CD25, and FOXP3^RFP^. Recipient BALB/c mice were imaged using IVIS 200 three times a week. Recipient BALB/c mice were also monitored for (B) changes in body weight, and (C) clinical score, and (D) survival for more than 40 days post BMT. For body weight changes and clinical score, one representative of two independent experiments is shown (*n* = 3 mice/group for BM alone; *n* = 5 experimental mice/group for all five other groups). (E) Quantitated luciferase bioluminescence of tumour growth. (F) Mortality from GVHD and tumour during the experiment, as a percent of mice dead. Statistical analysis for survival and the clinical score was performed using the log‐rank test and one‐way ANOVA with Tukey's test, respectively, and analysis for weight changes was done using one‐way ANOVA with Tukey's test. One representative experiment out of 2 is shown for A, C‐E. B and F are a combination of two experiments, three‐ to five mice per group. Symbol meaning for *p* values are: ns, *p* > .05; * *p* ≤ .05; ** *p* ≤ .01; *** *p* ≤ .001; **** *p* ≤ .0001. Note: Control mouse is a recipient mouse given _TCD_BM only (group 1), used as a negative control for BLI (no bioluminescent tumour cells were given)

Next, we examined the phenotypic characteristics of these canTregs and ncTregs. Central Treg cells (cTregs, CD62L^+^ CCR7^+^ CD44^‐^) are naïve‐like cells which when activated become effector Treg cells (eTregs, CD62L^−^ CCR7^−^ CD44^+^). These eTregs (also defined as activated Tregs or effector memory Tregs) express KLGR1, CD103, CTLA‐4 and ICOS, which are important functional markers.^39^, ^40^ Effector Tregs have more suppressive ability compared to central naïve‐like Tregs. Published data have shown that cTreg and eTreg cells mostly rely on IL‐2 and ICOS for maintenance, respectively.^39^

Our data show that canonical Tregs from *Itk^–/–^* mice have significantly more eTregs (CD44^+^CD62L^–^) and fewer central naïve‐like Tregs (CD44^–^ CD62L^+^) than canonical Tregs from WT mice. Noncanonical Tregs showed a significantly more effector‐like phenotype compared to canonical Tregs, regardless of strain (WT or *Itk^–/–^*), and *Itk* ^–/−^ Tregs are also more eTreg‐like and exhibit a lower central naïve‐like phenotype compared to Tregs from WT mice, regardless of canonical or noncanonical status (Figure 2G and Figure S4A,B). To see whether the increase in effector Tregs from *Itk^–/–^* was related to the effector and memory phenotype of conventional T cells, we examined both CD4^+^ and CD8^+^ T cells from WT C57Bl/6 to ITK‐deficient mice. Our data showed that CD4^+^ T cells from ITK‐deficient mice had significantly more effector memory cells and less naïve cells compared to CD4^+^ T cells from WT C57Bl/6 mice, but we did not observe any significant differences between central memory cells from ITK‐deficient mice compared to WT C57Bl/6 mice. (Figure 2H; Figure S4C). These data suggest that the increase in FOXP3 phenotype could be due to the enhanced memory phenotype of CD4^+^ T cells. However, the fact that *Itk^–/–^* canTregs and ncTregs phenotypes were maintained post‐transplantation suggests that these are cells with a distinct phenotype. Our data also showed that there was no significant difference in effector memory or transitioning to effector memory cells in CD8^+^ T cells from ITK‐deficient mice compared to WT C57Bl/6 mice. However, as previously reported, we saw an increase in CD8^+^ T cells with a central memory phenotype from ITK‐deficient mice, and less naïve cells compared to WT mice (Figure 2H; Figure S4D).^18^, ^19^

Chemokines play an indispensable role in the trafficking of allogeneic donor T cells into the sites of inflammation during GVHD. Th1‐like cytotoxic T cells use the receptor CXCR3 to migrate to GVHD target organs like lungs, liver and gut.^41^ Adoptively transferring CXCR3‐expressing Tregs can significantly ameliorate acute GVHD in the liver, lung, and small intestines.^21^ This effect was due to an increase in migration of the CXCR3^+^ Tregs, and the Tregs maintained their function in the localized GVHD target organs for a longer time, resulting in the better suppressive ability.^21^ Our data revealed that a significantly higher proportion of noncanonical Tregs express CXCR3 compared to canonical Tregs, regardless of strain (WT or *Itk^–/^* ^–^) (Figure 2I; Figure S5A). A higher proportion of *Itk^–/^* ^–^ Tregs also expressed CXCR3 compared to WT Tregs regardless of canonical or noncanonical status (Figure 2I; Figure S5A), suggesting that both canonical and noncanonical *Itk^–/–^* Tregs may have better suppressive ability in attenuating acute GVHD compared to canonical or noncanonical Tregs from WT mice.

PD‐1 is a transmembrane molecule that is encoded by the Pdcd1 gene and plays a critical role in Treg suppressive ability.^42^ When we analyzed PD‐1 expression of Tregs from both *Itk^–/–^* and WT mice, we observed that a significantly higher proportion of ncTregs express PD‐1 compared to canTregs from *Itk^–/–^* and WT mice (Figure 2J and Figure S5B) We did not observe any differences in proportion of *Itk^–/–^* and WT canTregs that express PD‐1, suggesting that PD‐1 expression is associated with the noncanonical phenotype rather than by Itk signaling (Figure S5B).

As previously mentioned, ICOS plays an indispensable role in maintenance of eTregs, which have better suppressive ability compared to central naïve‐like Tregs.^8^, ^43^ We also found that a higher proportion of *Itk^–/–^* ncTregs express ICOS compared to *Itk* ^–/−^ or WT canTregs and WT ncTregs (Figure 2K; Figure S5C). A higher proportion of *Itk^–/–^* canTregs express ICOS compared to canTregs from WT mice, suggesting that the increased eTreg phenotype in *Itk^–/–^* mice may be maintained by increased ICOS (Figure 2K; Figure S5C). Furthermore, a lower proportion of *Itk^–/–^* canonical and noncanonical Tregs express CCR7 compared to WT Tregs, suggesting a more effector‐like Treg phenotype for *Itk* ^–/−^ cells (Figure 2L; Figure S5D). Altogether, these data suggest that ncTregs from *Itk^–/–^* mice may have better suppressive abilities compared to other Treg groups, and may be more suppressive than WT Tregs in preventing GVHD.

### 3.3 Noncanonical *Itk^–/–^* Tregs suppress GVHD but maintain GVL effects

We next sought to determine whether ncTregs from *Itk* ^–/–^ mice are actually suppressive, and whether they could ameliorate GVHD responses while maintaining GVL efficacy. We once again used an MHC‐mismatch allogeneic transplant model as described above (WT C57Bl/6 into BALB/c). Recipient mice were lethally irradiated and transplanted with 10 × 10^6^ T cell‐depleted bone marrow cells, and other cells as described further below. Recipient transplanted mice were examined for cancer clearance via tumour growth signals weekly using bioluminescence (with the IVIS system) as described.

The first group of recipient mice was transplanted with bone marrow alone as a control, and these mice all survived without developing GVHD (Figure 3A). The second group of recipient mice was allogeneically transplanted with bone marrow cells and also 1 × 10^5^ luciferase‐expressing B‐ALL tumour cells.^12^, ^22^ These mice did not develop GVHD, but by day 31, all recipient mice died of tumours (Figure 3A). The third group of recipient BALB/c mice was transplanted with T cell‐depleted bone marrow and additionally 1 × 10^5^ luciferase expressing B‐ALL cells. This group of animals was transplanted with 1 × 10^6^ MACS‐purified CD8^+^ T cells from WT C57Bl/6. These mice were able to clear cancer cells, but developed acute GVHD and died within 2 weeks of transplantation (Figure 3A).The fourth group of recipient BALB/c mice was transplanted with bone marrow cells and challenged with 1 × 10^5^ luciferase expressing B‐ALL. This group of mice was also injected with 1 × 10^6^ WT CD8^+^ T cells, and 0.5 × 10^6^ canonical Tregs sorted from WT mice (using CD25^+^ and FOXP3 identified using an IRES RFP (FOXP3^RFP^).^44^ These recipient BALB/c mice were able to clear B‐ALL‐*luc* cells but showed delayed development of GVHD, and had to be euthanized within 4 weeks of transplantation (Figure 3A). The fifth cohort of mice was transplanted with bone marrow cells and 1 × 10^6^ CD8^+^ T from WT C57Bl/6, challenged with luciferase expressing B‐ALL cells, and further treated with 0.5 × 10^6^ canonical Tregs sorted from ITK‐deficient mice by CD25^+^ FOXP3^RFP^. This group of mice cleared the cancer cells, and only one out of six animals had to be euthanized due to GVHD (Figure 3A). The sixth group of mice was transplanted with bone marrow cells and with 1 × 10^6^ CD8^+^ T cells from WT C57Bl/6 mice. This group of mice was further challenged with tumour cells as described. This group of mice was also transplanted with 0.5 × 10^6^ noncanonical Tregs sorted from ITK‐deficient mice by CD25^–^ FOXP3^RFP^. These animals were able to clear the tumour cells, and only one out of seven animals had to be euthanized due to GVHD (Figure 3A). Unfortunately, we were not able to sort sufficient noncanonical WT Tregs for analysis.

We did not observe any statistical differences in survival, weight loss or clinical score for recipient mice that were untreated versus treated with canonical Tregs from WT mice (group 3 vs. group 4) (Figure 3B–D). We did observe statistical differences in survival and weight loss in the groups that received *Itk^–/–^* canTregs and ncTregs compared to the recipients of WT canTregs (group 5 and 6 vs. group 4) (Figure 3B–D). Clinical scores were significantly improved in the group that received *Itk^–/–^* ncTregs compared to the WT canTregs group (group 6 vs. group 4) (Figure 3C). Even though recipient BALB/c mice given *Itk^–/–^* canonical Tregs initially showed minimal signs of GVHD, similar to those given *Itk^–/–^* noncanonical Tregs, we observed increased signs of GVHD after 35 days (Figure 3C).

On day 17 and day 24 post‐transplant, bioluminescent imaging signals in mice from Group 2 were saturated, so we had to exclude those numbers. Despite the inability to perform statistical analysis in these groups, the trend in tumour growth was still apparent (Figure 3E). All groups which received WT CD8^+^ T cells cleared the tumour cells, while mice not given T cells died from tumour burden (Figure 3E,F). Additionally, mice not given Tregs died of GVHD, while mice given Tregs (especially from *Itk^–/–^* donors) had better survival (Figure 3F). Our findings demonstrate that ncTregs from *Itk* ^–/−^ mice suppress GVHD mediated by conventional T cell damage without affecting GVL responses against primary tumour cells. Itk deficiency also improves the suppressive function of canonical Tregs during GVHD.

### 3.4 Noncanonical *Itk^–/–^* Tregs suppress serum level inflammatory cytokine production

Inflammatory cytokines are one of the major causes of GVHD development following allogeneic transplantation of donor T cells.^45^ To assess suppression of inflammatory cytokine production by noncanonical Tregs from *Itk^–/–^* mice, we employed the allogeneic transplant model as described above (B6 into BALB/c) to cause GVHD. CD8^+^ T cells from WT C57Bl/6 mice were transplanted into recipient BALB/c mice. These mice were also treated with either canonical or noncanonical Tregs. Recipient mice were euthanized at day 7 post‐transplant; recipient animals were euthanized and serum levels of proinflammatory cytokines were examined using multiplex ELISA.

**FIGURE 4:**
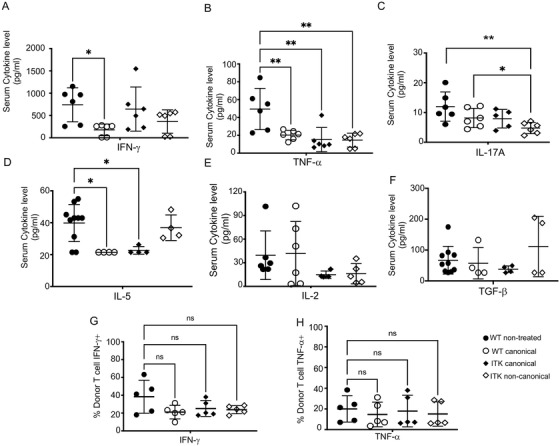
Noncanonical *Itk* ^–/−^ Tregs suppress serum level inflammatory cytokine production by donor T cells. (A‐F) 1 × 10^6^ purified WT CD3^+^ T cells were transplanted with _TCD_BM into irradiated BALB/c mice. At day 7 post allo‐HSCT, recipient BALB/c were euthanized and serum cytokines (IFN‐γ, TNF‐α, IL17A, IL‐5, IL‐2 and TGF‐β were determined by multiplex ELISA. (G) IFN‐γ expression by donor CD8^+^ T cells taken from recipient spleen 7 days post‐transplant. (H) TNF‐α expression by donor CD8^+^ T cells taken from recipient spleen 7 days post‐transplant. Combined data from two independent experiments is shown for cytokine restimulation. Statistical analysis was performed using one‐way ANOVA with Tukey's test, *p* value presented with each figure. Symbol meaning for *p* values are: ns, *p* > .05; * *p* ≤ .05; ** *p* ≤ .01; *** *p* ≤ .001; **** *p* ≤ .0001. One experiment's data are shown as representative from three independent experiments for serum ELISA

Our data showed that recipient (B6 into BALB/c) mice treated with WT canTregs have significantly less IFN‐γ in the serum compared to mice not given Tregs (Figure 4A), but no differences in the serum IFN‐γ levels in mice treated with *Itk* ^–/−^ canTregs or ncTregs (Figure 4A). We found that recipient mice treated with either *Itk* ^–/−^ or WT canTregs and ncTregs showed significantly less TNF‐α from the serum when compared to mice that were not treated with Tregs (Figure 4B). Next, we found that the recipient mice treated with ncTregs from *Itk* ^–/−^ mice had a significant decrease in IL‐17A in serum compared to mice not treated with Tregs (Figure 4C). Our data showed recipient mice treated with canTregs from either ITK‐deficient mice or WT C57Bl/6 mice showed significantly less IL‐5 in serum than non‐treated recipients (Figure 4D). However, we did not see significant differences in the serum levels of IL‐4, IL‐6, IL‐22, IL‐17F, IL‐13, IL‐9, IL‐10 (data not shown) and IL‐2 or TGF‐β (Figure 4E,F).

We also obtained lymphocytes from the secondary lymphoid organs (spleen and inguinal lymph nodes) of recipients at 7 days post‐transplant. We stimulated spleen cells with TCR stimulation by anti‐CD3 and anti‐CD28 for 6 h (Figure 4G,H), with a portion of cells not stimulated as a control. We cultured these ex *vivo* cells with anti‐CD3 and anti‐CD28 with GolgiPlug. These ex *vivo* cells were stained for H‐2K^b^ (expressed on donor cells), CD3, CD8 and CD4. Using flow cytometry, we examine these cells for inflammatory cytokine production, including TNF‐α and IFN‐γ. We did not find any significant difference in IFN‐γ and TNF‐α production by WT donor CD8^+^ T cells in animals treated with WT or *Itk^–/–^* canTregs or ncTregs (Figure 4G,H), although these cells are stimulated *ex vivo*, which may not reflect the situation in vivo under Treg suppression.

We observed significant differences in IFN‐γ and TNF‐α production. These changes in serum could potentially be explained by a decrease in proliferation of donor conventional T cells in vivo. Therefore, treatment with Tregs from WT or *Itk* ^–/−^ mice altered the serum levels of several important cytokines following allotransplant.

### 3.5 Noncanonical *Itk^–/–^* Tregs suppress donor T cell proliferation in vivo, resulting in less damage to GVHD target organs

**FIGURE 5:**
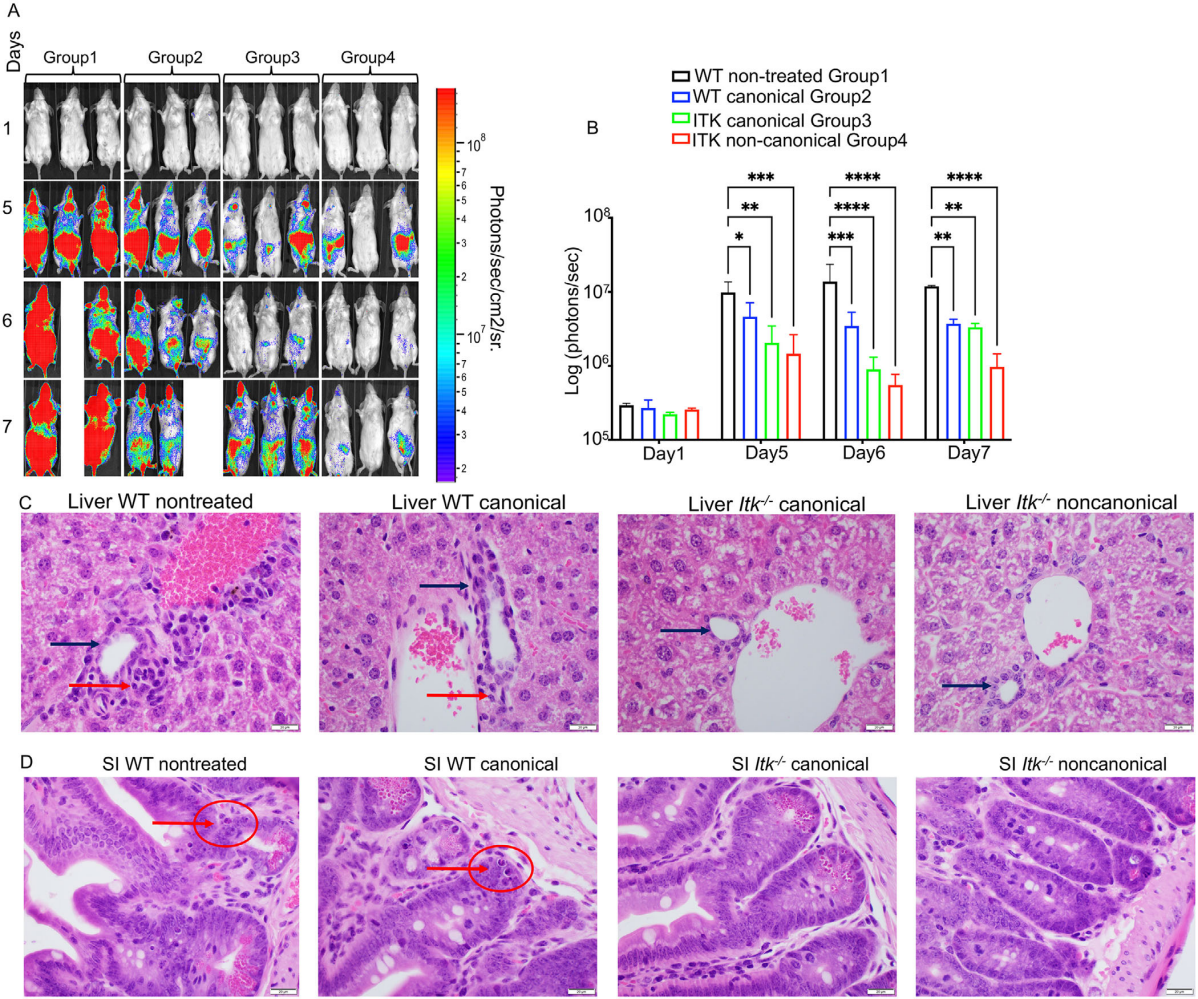
Noncanonical *Itk* ^–/−^ Tregs suppress donor T cell proliferation in vivo, resulting in less damage to GVHD target organs. (A) BALB/c recipient mice for all groups were lethally irradiated and transplanted with 10 × 10^6^ T cell‐depleted bone marrow cells and 1 × 10^6^ WT‐*luc* ^+^ CD8^+^ T cells (donor T cells expressing luciferase). *Group 1* recipient mice were not given any additional cells (non‐treated). *Group 2* BALB/c recipient mice were treated with FACS sorted canonical Tregs from WT C57Bl/6 mice. *Group 3* BALB/c recipient mice were treated with FACS sorted canonical Tregs from *Itk* ^–/−^ mice. *Group 4* BALB/c recipient mice were treated with FACS sorted noncanonical Tregs from *Itk* ^–/−^ mice. Recipient BALB/c mice were imaged using IVIS 50 every day for 7 days post‐transplant in order to track the transplanted WT‐*luc* ^+^ CD8 T cells' proliferation in the different treatment groups. (B) Quantification of luciferase bioluminescence, representing CD8‐*luc* ^+^ donor T cell proliferation. Statistical analysis was performed using one‐way ANOVA with Tukey's test, one experiment is shown. (C‐D) BALB/c mice were transplanted as described in (A), except the WT CD8 T cells were from WT mice (not WT luc). At day 7 post‐transplantation, recipient mouse livers and small intestines were obtained, sectioned, and stained with H&E. Representative photos or recipient organs for each treatment group are shown. Statistical analysis was performed using a Chi‐square test and Kruskal Wallis test followed by Dunn's multiple comparison test. *p* Value presented with the figure. Symbol meaning for *p* values are: ns, *p* > .05; * *p* ≤ .05; ** *p* ≤ .01; *** *p* ≤ .001; **** *p* ≤ .0001. One experiment is shown as a representative from two independent experiments

To examine whether these ncTregs from ITK‐deficient mice might suppress GVHD caused by conventional CD8^+^T cells in the allogeneic transplant model, we specifically examined donor CD8^+^T cell proliferation. We used an allogeneic transplant model as described above. CD8^+^T cells from luciferase‐expressing C57Bl/6 mice^12^, ^13^ were transplanted into irradiated BALB/c mice as donor cells.^12^, ^13^ Recipient animals were then examined post‐transplant for luciferase expression as a means to examine CD8^+^ T cell proliferation, measured by bioluminescence (Figure 5A). One group of BALB/c recipients was allogeneically transplanted with T cell‐depleted bone marrow cells from non‐luc WT C57Bl/6 mice. In addition, we transplanted this group of animals with 1 × 10^6^ CD8^+^ T cells from WT *luc* mice (group 1). This group of mice exhibited an increase in luc+ donor CD8^+^ T cell proliferation. (Figure 5A,B). The second cohort of recipient mice was transplanted with T cell‐depleted bone marrow cells from non‐luc WT C57Bl/6 mice. In addition, we transplanted this group of animals with 1 × 10^6^ CD8^+^ T cells from WT *luc* mice, and further treated them with 0.5 × 10^6^ canonical Tregs FACS sorted from C57Bl/6 FOXP3^RFP^ WT mice by CD25^+^ FOXP3^RFP^. These animals showed that treatment with 0.5 × 10^6^canonical Tregs led to a reduction in conventional CD8^+^T cell proliferation (Figure 5A,B). The third cohort of recipient mice was treated with T cell‐depleted bone marrow cells from non‐luc WT C57Bl/6. In addition, we transplanted this group of animals with 1 × 10^6^ CD8^+^ T cells from WT *luc* mice and treated them with 0.5 × 10^6^ canTregs sorted from *Itk* ^–/−^ FOXP3^RFP^ mice by CD25^+^ FOXP3^RFP^ (Figure 5A,B). The last cohort of recipient BALB/c mice was treated with T cell‐depleted bone marrow cells from non‐luc WT C57Bl/6. In addition, we transplanted this group of animals with 1 × 10^6^ CD8^+^ T cells from WT *luc* mice. Additionally, this group of mice was treated with 0.5 × 10^6^ ncTregs sorted from *Itk* ^–/−^ FOXP3^RFP^ mice by CD25^+^ FOXP3^RFP^. This group of animals showed a significantly higher reduction in donor CD8^+^ T cell proliferation (Figure 5A,B).

All transplanted mice were monitored for bioluminescence (representing donor T cell proliferation) every day for 7 days (Figure 5A,B). Day 1 post transplantation, there were no differences among any group. However, on day 5 and day 7, we observed differences among groups. Total BLI was used to measure the reduction in donor CD8^+^ T cell proliferation (Figure 5B). The noncanonical *Itk* ^–/−^ Tregs demonstrated the most robust decrease in donor cell proliferation compared to the other groups (Figure 5B). Previous work suggested that a 1:1 ratio of Tregs to conventional T cells is needed to see a significant reduction in donor T cell proliferation.^46^ Here, our data support this fact, as the use of a 1:2 Treg:Tconv ratio for canTregs (WT or *Itk^–/–^*) allowed for initial suppression of donor T cell proliferation (Figure 5A,B), but GVHD was not permanently ameliorated (Figure 3). However, treatment with *Itk^–/–^* ncTregs even in a 1:2 Treg:Tconv ratio was able to reduce donor T cell proliferation early on, as well as persistently alleviate acute GVHD signs (Figure 3). These data demonstrate that *Itk^–/–^* Tregs may have equal or superior suppressive abilities compared to WT canTregs in this model.

We also repeated the above allogeneic transplant experiments as described to perform histology analyses. We transplanted recipient mice with 1 × 10^6^ CD8^+^ T cells from WT C57Bl/6 mice. Recipient mice were either non‐treated or treated with 0.5 × 10^6^ FACS‐sorted Tregs from WT FOXP3^RFP^ or *Itk^–/–^* FOXP3^RFP^ mice on the same day as the transplant. Canonical Tregs were obtained from either WT C57Bl/6 or ITK‐deficient mice, while noncanonical Tregs were obtained from *Itk* ^–/−^ mice only. Day 7 post‐allogeneic transplantation, we euthanized recipient animals and isolated target organs as sites of inflammation. We stained these GVHD target organ tissues for H&E, and we saw significant donor T cell infiltration into liver and small intestine (SI) from animals not treated with Tregs (Figure 5C,D). In the WT non‐treated group, liver histology shows significant changes of acute GVHD: interlobular bile duct epithelium (black arrow) is infiltrated and destroyed by predominantly lymphocytes (red arrow) with no marked fibrosis. In the WT canonical group, bile duct (black arrow) damage is less significant with fewer infiltrating lymphocytes (red arrow). In *Itk* ^–/−^ groups, both canonical and noncanonical treatments show significant improvement of GVHD in the liver: the interlobular bile ducts are normal‐appearing and free of inflammatory cells (Figure 5C; Figure S6).

Both WT non‐treated and WT canonical Treg‐treated groups show mild acute GVHD in small intestine with the features of occasional apoptotic bodies (red arrow and red circle) without necrosis or crypt dropout. Groups that were treated with canTregs or ncTregs from *Itk^–/–^* mice show no GVHD effects in the small intestine (Figure 5D; Figure S6). Our data suggest that noncanonical Tregs from Itk‐deficient mice have equal or greater suppressive ability than canonical Tregs from WT or Itk‐deficient mice. These noncanonical Tregs can suppress donor CD8^+^ T cell expansion in an in vivo model of GVHD following allo‐HSCT, supporting their utility as a treatment for T cell‐mediated disorders.

### 3.6 Noncanonical *Itk^–/–^* Tregs have different gene expression patterns than canonical Tregs

Next, we wanted to examine whether noncanonical Tregs from Itk‐deficient mice have a unique genetic program compared to WT or Itk‐deficient canonical Tregs. To do this, we obtained MACS‐purified CD4^+^ T cells (three replicates for each group), and cells were further purified using the flow sorter. We obtained FACS‐sorted WT canonical or ITK‐deficient canonical and noncanonical Tregs using FOXP3^RFP^, CD4, CD3 and CD25 as markers. The noncanonical WT‐FOXP3^RFP^ Treg percentage was so small that we could not sort enough cells for downstream applications, so the WT ncTregs group was excluded. RNA was then extracted from sorted cells, the cDNA libraries were prepped using the SMART‐Seq HT kit (Takara Bio) and samples were sequenced on an Illumina NovaSeq 6000 sequencer.

**FIGURE 6:**
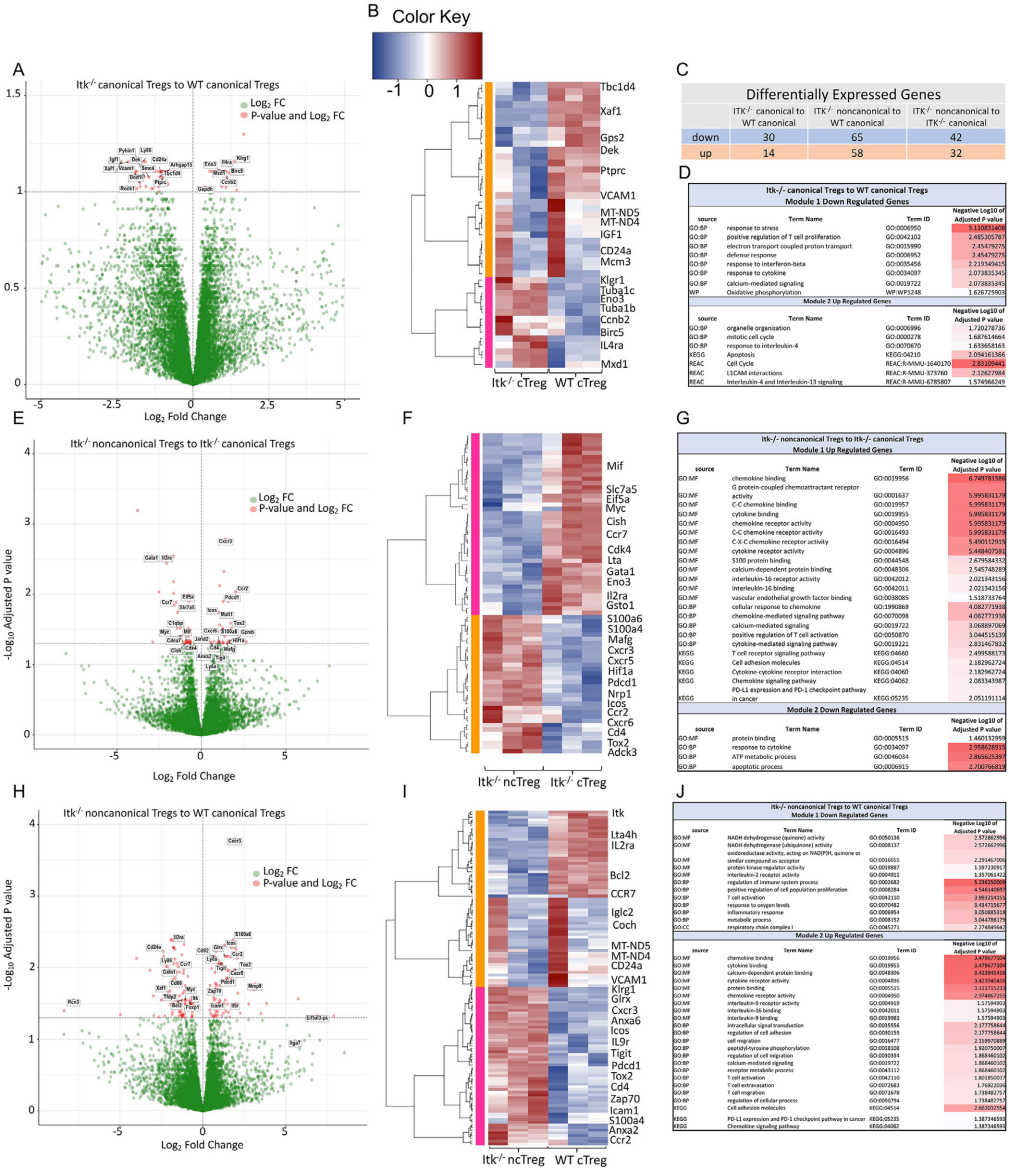
Noncanonical *Itk* ^–/−^ Tregs have different gene expression patterns than canonical Tregs. (A) Volcano plot showing differentially expressed genes (FDR ≤ .1) between *Itk^–/–^* canonical and WT canonical Tregs. (B) Hierarchical clustering of genes and heatmap illustrating expression of genes compared between *Itk^–/–^* canonical and WT canonical Tregs. All replicates are shown (*n* = 3) for each group. Modules are identified by numbers and by colour distinguishing up‐ and downregulated genes in groups. (C) Table showing the number of up or downregulated DEGs between groups for each of three separate comparisons made. (D) GO annotation analysis table of up‐ and downregulated genes between *Itk^–/–^* canonical and WT canonical Treg groups. (E) Volcano plot showing differentially expressed genes (FDR≤ .05) between *Itk^–/–^* noncanonical and *Itk^–/–^* canonical Tregs. (F) Hierarchical clustering of genes and heatmap illustrating expression of genes compared between *Itk^–/–^* noncanonical and *Itk^–/–^* canonical Tregs. (G) GO annotation analysis table of up‐ and downregulated genes between *Itk^–/–^* noncanonical and *Itk^–/–^* canonical Treg groups. (H) Volcano plot showing differentially expressed genes (FDR≤.05) between *Itk^–/–^* noncanonical and WT canonical Tregs. (I) Hierarchical clustering of genes and heatmap illustrating expression of genes compared between *Itk^–/–^* noncanonical and WT canonical Tregs. (J) GO annotation analysis table of up‐ and downregulated genes between *Itk^–/–^* noncanonical and WT canonical Treg groups

DEGs between each group of Tregs were averaged by group first, and then we determine the gene co‐regulation by hierarchical clustering using Pearson correlation with a grouping cutoff (k) of 3.^47^ Each generated module was created as heatmap and represents a group of altered genes that were up‐ or downregulated in the corresponding comparison. Analysis of the canTreg cell populations from WT and ITK‐deficient mice identified as 44 differentially expressed genes (DEGs; FDR ≤ .1) (Figure 6A–C). Of these DEGs, 30 genes were downregulated (including Gps2, Ptprc, Vcam1, MT‐ND5, MT‐ND4 and IGF1) and 14 genes were upregulated (including Il4ra, Klrg1, Birc5, Ccnb2 Eno3) for *Itk* ^–/−^ canTregs versus WT canTregs. (Figure 6A,C). Genes like Birc5, Ccnb2, Mcm3, Tuba1b and Tuba1c, which play a role in cell cycle, were also upregulated in Itk^–/–^ canTregs compared to WT canTregs (Figure 6A,B), suggesting that Itk^–/–^ canTregs may be proliferating more compared to WT canTregs. GO annotation analysis of down‐regulated genes revealed that these genes play a role in responses to stress, regulation of T cell proliferation, electron transport chain, oxidative phosphorylation and other pathways, and genes which were upregulated are involved in organelle organization, cell cycle, L1cam interaction and other pathways (Figure 6D).

We identified 74 genes that are differentially expressed (DEGs; FDR ≤ .05) among *Itk^–/–^* noncanonical Tregs and *Itk^–/–^* canonical Tregs (Figure 6C,E,F). Of these, 42 genes were downregulated (including Slc7a5, Myc, Ccr7, Lta, Gata1 and Il2ra), and 32 genes were upregulated (including CXCR3, CXCR5, CXCR6, Hif1a, Pdcd1, ICOS, Ccr2, CD4, Tox2, Adck3 and NRP1) (Figure 6E). The Il2ra gene (coding for CD25) was downregulated only in *Itk* ^–/−^ ncTregs, which served as a control showing that the ncTreg sample included Tregs lacking expression of CD25. Chemokine receptors like CXCR5, CXCR6, CXCR3 and CCR2 were also highly upregulated in *Itk* ^–/−^ ncTregs compared to *Itk* ^–/−^ canTregs, suggesting that *Itk* ^–/−^ ncTregs cells could be highly differentiated Tregs with tissue‐homing receptors. *Itk^–/–^* noncanonical Tregs upregulated the NRP1 gene compared to WT canTregs, suggesting that these ncTregs are thymus‐derived natural Tregs.^47^, ^48^ Our RNA‐seq analysis showed that *Itk* ^–/−^ ncTregs downregulate EIf5a1, and it is known that elF5A inhibition enriches Treg populations (Imam et al., 2019). Also, the Slc1a5 gene was downregulated in *Itk^–/–^* ncTregs, and previous work has shown that *Slc1a5^–/–^* mice are resistant to induction of T cell‐dependent autoimmunity.^48^ Slc1a5 gene is a negative regulator of Treg function, leading to enhanced activity in the Slc1a5 KO mice. There were also a number of other genes that were differentially expressed in *Itk^–/–^* ncTregs compared to Itk^–/–^canTregs (Figure 6F).

The alteration of genes like CXCR3, ICOS, CCR7 and Pdcd1 was expected based on functional data described earlier in the manuscript. GO annotation analysis showed that downregulated genes were involved in a number of different pathways like protein binding, metabolic processes and apoptosis. Genes found to be upregulated also play a role in a number of important pathways like chemokine binding, cytokine binding, chemokine receptor activity, IL‐16 binding and receptor activity, the TCR pathway, cell adhesion molecules and the PD1 pathway (Figure 6G).

For a final comparison between *Itk^–/–^* ncTreg and WT canTreg groups, analysis reveal 123 differentially expressed genes (DEGs; FDR≤ .05), with 65 genes being downregulated (including Itk, Il2ra, Bcl2, Ccr7, MT‐ND4, MT‐ND5 and CD24a) and 58 genes being upregulated (including VCAM1, Klrg1, Glrx, Cxcr3, Anxa6, Anxa2, Icos, IL9r, Tigit, Pdcd1, Tox2, CD4, Zap70, Ccr2 and Icam1) in *Itk^–/–^* ncTregs compared to WT canTregs (Figure 6C,H,I). The Itk gene was downregulated in *Itk^–/–^* samples, and Il2ra (CD25) was downregulated only in *Itk* ^–/−^ ncTregs, which both served as controls showing that gene expression for the grouped samples was as expected for these genes. T cell activation regulator and effector molecules like Klrg1, Glrx, Itgb1 and ICOS^37^ were differentially expressed by Itk^–/–^ ncTregs (Figure 6I). CCR7, which is a naïve Treg cell marker,^39^ was downregulated in Itk^–/−^ ncTregs, which correlates with the effector phenotype of those Tregs (Figure 6I). Chemokine receptors like CXCR3 and CCR2 were also highly upregulated in *Itk^–/–^* ncTregs, suggesting that these cells could be highly differentiated Tregs with tissue‐homing receptors (Figure 6F,I). Genes that play a role in cell adhesion were also highly upregulated in *Itk^–/–^* ncTregs, including CD4, ICAM1, Itga7, Itgb1 and Pdcd1. Bcl‐2, which is an anti‐apoptotic marker, was differentially downregulated in *Itk^–/–^* ncTregs (Figure 6I). It has been previously shown that inhibiting Bcl‐2 with a small molecule leads to the Treg‐dependent alleviation of acute GVHD.^49^ On the other hand, Itk^–/–^ ncTregs also have higher expression of TIGIT compared to other Tregs (Figure 6I). It has been previously shown that TIGIT‐expressing Tregs are a functionally distinct Treg cell subset with a more suppressive activated phenotype,^3^ which are able to suppress pro‐inflammatory T helper 1 (Th1) and Th17 cells.^50^ This suggests that noncanonical Tregs from *Itk* ^–/−^ mice may have a more suppressive Treg genetic program. GO annotation analysis also revealed that genes that were upregulated in *Itk^–/–^* ncTregs compared to WT canTregs play a role in chemokine and cytokine binding, chemokine receptor activity, IL‐9 binding and receptor activity, IL‐16 binding, regulation of cell adhesion and migration, T cell activation, extravasation, migration, PD1 expression and other pathways. Downregulated genes were involved in oxireductase activity, IL‐2 receptor activity, inflammatory response, metabolic process and others (Figure 6J).

**FIGURE 7:**
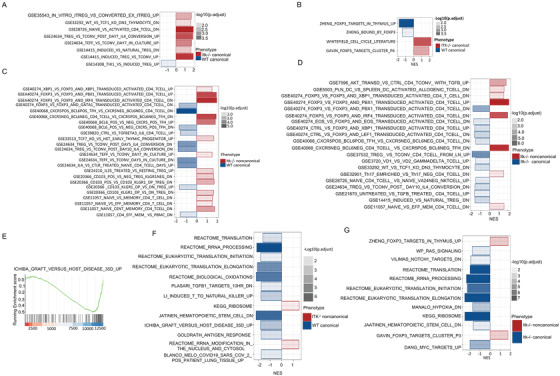
Gene set enrichment analysis of the WT or *Itk* ^–/−^ Treg groups. (A) Gene Set Enrichment Analysis (GSEA) results of *Itk^–/–^* canTregs versus WT canTregs using MSigDB C7 (immunological) gene sets. Negative normalized enrichment score (NES) is an indicator of downregulation and positive NES is an indicator of upregulation of the genes in the corresponding pathway. Colour specifies the group (*Itk^–/–^* canTregs or WT canTregs) in which expression is enriched; colour transparency indicates the negative Log10 of adjusted *p* value. (B) GSEA results of *Itk^–/–^* canTregs versus WT canTregs using MSigDB C2 (curated) gene sets. (C) GSEA results of *Itk^–/–^* ncTregs versus WT canTregs using MSigDB C7 (immunological) gene sets. (D) GSEA results of *Itk^–/–^* ncTregs versus *Itk^–/–^* canTregs using MSigDB C7 (immunological) gene sets. (E) Running enrichment score (ES) for the “ICHIBA_GRAFT_VERSUS_HOST_DISEASE_35D_UP” pathway genes, comparing *Itk^–/–^* ncTregs to WT canTreg. The ES for the pathway is defined as the peak score furthest from zero, with a negative ES meaning enrichment in the WT canTregs group. (F) GSEA results of *Itk^–/–^* ncTregs versus WT canTregs using MSigDB C2 (curated) gene sets. (G) GSEA results of *Itk^–/–^* ncTregs versus *Itk* ^–/−^ canTregs using MSigDB C2 (curated) gene sets

We also performed GSEA analysis (gene set enrichment analysis) using the C7 (immunological) and C2 (curated) pathway collections from Molecular Signatures Database (MSigDB). Pathway titles ending in “_DN” are lists of genes downregulated in the listed comparison, while pathways ending in “_UP” are upregulated. We then took the lists of genes in these comparisons and identified whether they were enriched in our Treg populations. We observed a small number of pathways that were significantly altered between *Itk^–/–^* canTregs and WT canTregs in both C2 and C7 MSigDB collections. This was expected considering the number of DEGs and FDR values between the *Itk^–/–^* canTregs and WT canTregs groups (Figure 7A,B). Our data also revealed that the genes that were downregulated between Naïve to Memory (GSE11057_NAIVE_VS_MEMORY_CD4_T_CELL_DN), Naïve to Central memory (GSE11057_NAIVE_CENT_MEMORY_CD4_TCELL_DN) and Naïve to Effector memory cells (GSE11057_NAIVE_VS_EFF_MEMORY_CD4_T_CELL_DN) were significantly enriched within the *Itk^–/–^* ncTregs group when we compared them with the WT canTreg group (Figure 7C). The same Naïve to Effector memory pathway was also significantly enriched in the *Itk^–/–^* ncTregs group when compared with the *Itk^–/–^* canTreg group (Figure 7D), suggesting that *Itk* ^–/−^ ncTregs may have more effector memory properties, which correlates with our flow cytometry data and DGE results. Interestingly, we also observed that a number of Tfr (T follicular regulatory) pathways were significantly enriched in *Itk^–/–^* ncTregs compared to *Itk^–/–^* canTregs, suggesting that *Itk^–/–^* ncTregs may have Tfr properties (Figure 7D).

When we interrogated the *Itk^–/–^* ncTregs versus WT canTregs in the MSigDB C2 (curated) collection, we observed that the genes upregulated in a hepatic GVHD on day 35 pathway (ICHIBA_GRAFT_VERSUS_HOST_DISEASE_35D_UP) were enriched in WT canTregs (Figure 7E,F). Those genes were downregulated in the *Itk^–/–^* ncTregs group, with a normalized enrichment score (NES) of −1.76 and −1.75, respectively (Figure 7E,F). We did not see differences in the GVHD pathway when we compared canTregs from WT and *Itk^–/–^* mice (Figure 7A,B), or when we compared ncTregs and canTregs from *Itk^–/–^* mice using the C2 curated collection (Figure 7G). This suggests that the effects on GVHD pathway may require both loss of Itk and the ncTreg phenotype at the genetic level. These are examples of pathways that are involved in different groups of Tregs from *Itk^–/–^* and WT mice. Important pathways that are related to our study are illustrated in Figure 7 in more detail.

### 3.7 The inhibitory peptide SLP76pTYR enhances Treg development and suppresses proinflammatory cytokine production in healthy human and GVHD patient samples

**FIGURE 8:**
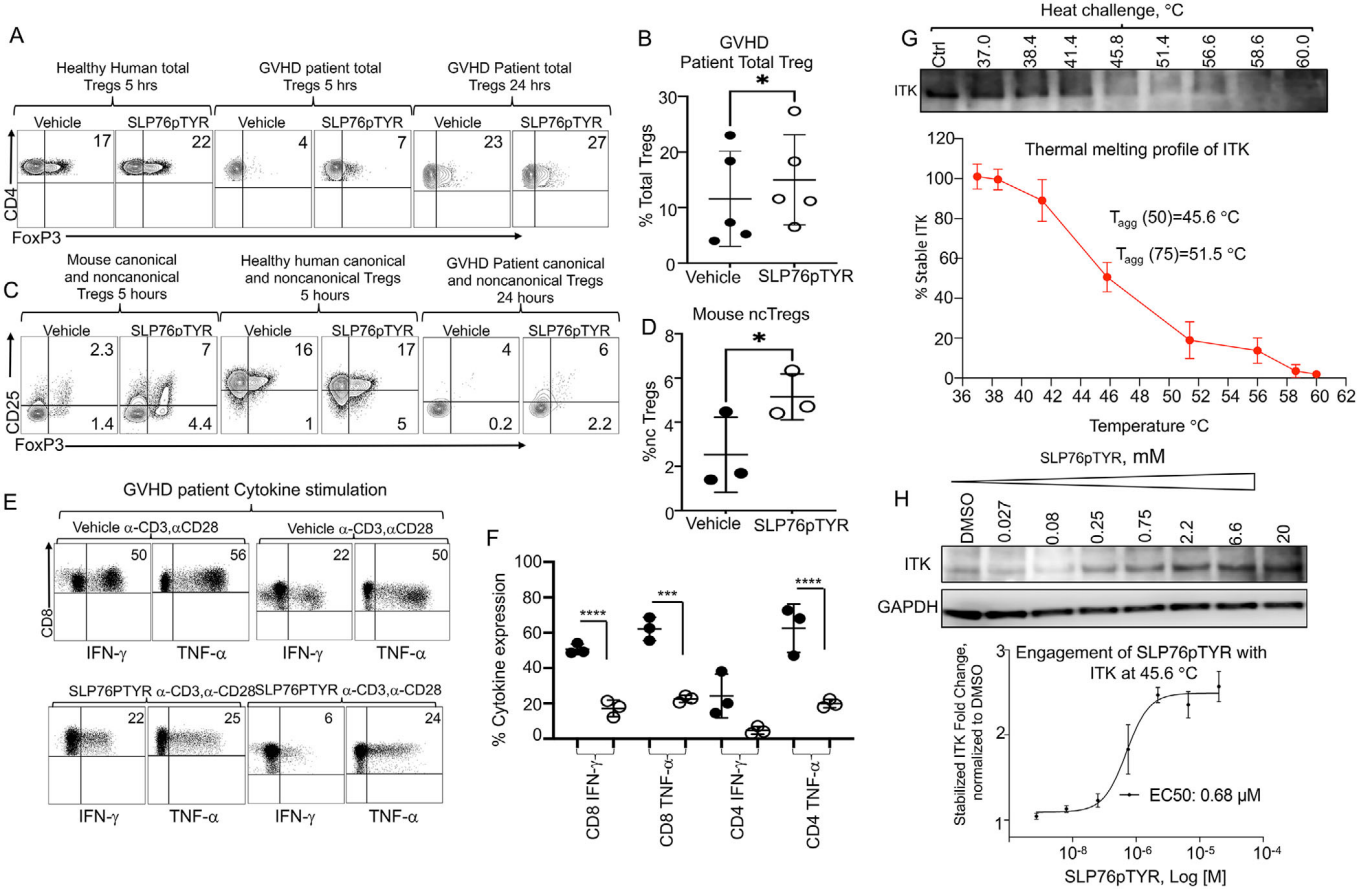
Disruption of Itk/SLP76 Y145 signaling enhanced FOXP3 expression and decreased proinflammatory cytokines in healthy human and GVHD patient samples. Peripheral blood mononuclear cells (PBMCs) of healthy donors or from GVHD patient donors, or mouse CD3 T cells were isolated from the spleens of naive WT C57Bl/6 mice. For Treg cell culture, cells were resuspended in media and stimulated with anti‐CD3 in the presence of Polybrene and SLP76pTYR or vehicle. Cells were cultured for 5 to 24 h, then stained for Treg markers. (A) CD4^+^ FoxP3^+^ cell percentage in 5‐ or 24‐h SLP76pTYR‐ or vehicle‐treated healthy human or GVHD patient PBMCs. (B) Quantification of five independent experiments of (A). (C) Canonical (CD4^+^ CD25^+^ FoxP3^+^) and noncanonical (CD4^+^ CD25^–^ FoxP3^+^) Treg percentage in SLP76pTYR or vehicle‐treated mouse T cells, healthy human PBMCs, and GVHD patient PBMCs. (D) Quantification of noncanonical Tregs in mice which were treated with SLP76pTYR or vehicle alone, from three independent experiments. (E) Human GVHD PBMC samples were stimulated with anti‐CD3/anti‐CD28 and treated with vehicle or SLP76pTYR, then cultured in the presence of GolgiPlug for 6 h. Cells were then stained for IFN‐γ and TNF‐α and analyzed by flow cytometry for CD8^+^ and CD4^+^ T cells. (F) Quantification of GVHD patient IFN‐γ and TNF‐α expression in CD8^+^ and CD4^+^T cells treated with vehicle or SLP76pTYR, from three independent experiments. (G‐H) CETSA was performed using fresh cell lysate from purified cultured mouse T cells, prepared in non‐denaturing buffer. Cell lysate was dispensed into a 96‐well PCR plate in the above medium (approx. 10 000 cells/well/50 μL), then was subjected to a temperature gradient (37‐60°C) for 20 min. Subsequently, centrifugation was performed at 14 000 rpm to sediment the unstable protein content. Supernatant was collected and an SDS‐PAGE gel was run, and immuno‐detection was performed for ITK using the corresponding primary antibody. Band intensity was quantified on a LI‐COR C‐Digit Blot Scanner, and the *T*_agg_(50) and *T*_agg_(75) values were calculated for ITK (G). In a subsequent run (H), fresh lysates from purified mouse T cells were treated at various doses with threefold dilutions (20, 6.6, 2.2, 0.75, 0.25, 0.08 and 0.027 μM) of the peptide SLP76pTYR or the DMSO control, for 1 h. Samples were then subjected to heat challenge at *T*_agg_(50) for 20 min, and unstable protein was removed by a centrifugation step. Following an immuno‐blotting step, bands of remaining stable ITK were quantified, normalized to loading control and plotted using GraphPad Prism software. EC50 values of engagement for both compounds with ITK were subsequently calculated. Statistical analysis was performed using one‐way ANOVA, *p* value presented with each figure. Symbol meaning for *p* values are: ns, *p* > .05; * *p* ≤ .05; ** *p* ≤ .01; *** *p* ≤ .001; **** *p* ≤ .0001. One experiment is shown as a representative from 3 independent experiments, for statistics data from 2–3 independent experiments pooled

We recently developed a specific inhibitor, SLP76pTYR, that can disrupt SLP76 and ITK signaling by preventing the SH2 domain of Itk from docking onto SLP76 at the tyrosine 145 position.^22^ Previously, we also showed that the SLP76pTYR peptide can enhance the expansion of murine Tregs.^22^ To determine whether the SLP76pTYR peptide can also enhance Treg development in human cells, PBMCs from healthy humans and from GVHD patients were cultured with our inhibitor or vehicle alone as a control in the presence of anti‐CD3 (clone OKT3) and polybrene (5 ug/ml) for 5 to 24 h. We saw a significant increase in FoxP3^+^ CD4^+^ T cells in GVHD patient primary cells treated with SLP76pTYR peptide, and a trend towards increased production in healthy human treated cells (Figure 8A,B; Figure S7A).

In order to determine whether the SLP76pTYR peptide can also enhance ncTreg phenotype in primary mouse and human cells, mouse primary T cells and healthy human or GVHD patient PBMCs were cultured with our inhibitor SLP76pTYR or vehicle alone as a control, and stimulated with anti‐CD3 and polybrene for 5 to 24 h. We observed a trend towards increased production of ncTregs in mouse, healthy human and GVHD patient primary cells treated with SLP76pTYR peptide (with significance in mouse cells) (Figure 8C,D; Figure S7B,C, as well as a trend towards increased production of canTregs for mouse and GVHD patients’ cells treated with peptide (Figure 8C; Figure S7D,E). Even though we observed that mouse T cells treated with SLP76pTYR showed a trend towards increased canTregs, the effect was not significant (Figure S7D). Healthy human T cells treated with SLP76pTYR did not show a significant increase in canTregs (Figure S7F), and we observed that healthy human cells treated with SLP76pTYR showed a trend towards increased ncTregs, but the effect was statistically non‐significant (Figure S7B). Our data showed that the SLP76pTYR inhibitor can affect noncanonical Treg populations in both mouse and human. Altogether, these data demonstrated that disrupting SLP76:ITK signaling increases Treg frequency. Our data suggest that our SLP76pTYR peptide has the potential to be used in a clinical setting to enhance Treg expansion for use in treating immune cell‐mediated diseases.

To assess the effect of SLP76pTYR on proinflammatory cytokine expression in GVHD patients, we isolated PBMCs from total blood of patients suffering with GVHD. Total cells were either stimulated with anti‐CD3 and anti‐CD28 and cultured with SLP76pTYR and polybrene, or stimulated PBMCs were cultured with vehicle alone and polybrene using GolgiPlug to prevent cytokine secretion. PBMCs were cultured in a 12‐well plate for 6 h. Cells were stained for IFN‐γ and TNF‐α and analyzed using flow cytometry. We have specifically gated on human CD4^+^ and CD8^+^ T cells. Our data show that GVHD patient cells stimulated with anti‐CD3/anti‐CD28 and cultured with SLP76pTYR showed significantly reduced production of IFN‐γ and TNF‐α from CD8^+^ T cells compared to patient cells cultured in the presence of vehicle alone (control) (Figure 8E,F). The stimulated cells in the GVHD patient cytokine suppression assay were compared to a non‐stimulated control, with or without SLP76pTYR peptide (Figure S8).

To examine whether the SLP76pTYR peptide induces apoptosis or causes non‐specific cell death, human T cells isolated from PBMCs from patient blood were cultured for 5 h with SLP76pTYR inhibitors or vehicle alone (control). We stained the cells for CD3, CD4, CD8 and Annexin V and LIVE/DEAD Near‐IR to detect apoptosis and cell death. We did not observe any significant differences in live or apoptotic cells between SLP76pTYR‐ and vehicle‐treated groups, for either CD4^+^ or CD8^+^ T cells (Figure S9A‐D). Similarly, we isolated splenocytes of WT C57Bl/6 mice and cultured them with SLP76pTYR and vehicle alone for 5 h, and post‐culture splenocytes were stained for CD3, CD4, CD8, Annexin V and LIVE/DEAD Near‐IR. We did not see any differences in cell death or apoptosis for mouse cells (Figure S10A–D). To further confirm that the peptide was not toxic to cells, we used mouse T cell line Yac‐1 cells, and B cells line A20 cells, which were transduced with GFP and luciferase,^24^ and we cultured them in the presence of SLP76pTYR. Cells were imaged by adding luciferin^24^ and imaged every hour from 0 up to 6 h (Figure S10E–H). We did not observe a significant reduction in bioluminescence of the cells that were cultured with SLP76pTYR peptide or vehicle alone, suggesting that the peptide does not have toxicity at this level in cell culture.

It was also important to determine whether the observed effects of the peptide were due to direct engagement of the SLP76pTYR peptide with ITK. In order to test cellular target engagement, in the first step, we utilized a thermal stability assay as described by Savitski et al.,^51^ to establish the thermal melting profile of ITK. We determined that the temperature of aggregation (T‐agg 50) of ITK was 45.6°C, at which 50% of ITK protein in the cell is denatured (Figure 8G). In the second step, we treated the cells with SLP76pTYR, and then subjected them to temperature challenge at T‐agg. The rationale of this methodology is that heat‐induced denaturation of ITK would be rescued by direct engagement with the ligand as has been described for other kinases. Direct engagement of the ligand will biophysically stabilize the target, and therefore its immunoblot band will become more intense. Denatured/unrescued target protein was removed by centrifugation prior to immunoblotting. Therefore, the stronger ITK bands observed in the immunoblot show that SLP76pTYR rescued the target from denaturation in a dose‐proportional way, with an EC50 of the 0.68 μM (Figure 8H). As a result of this direct engagement, SLP76pTYR is expected to block ITK from interacting with its partner proteins. These data demonstrate direct engagement of the SLP76pTYR peptide with ITK as an essential part of the mechanism of action in inhibiting cytokine expression by human T cells from GVHD patients.

## 4 DISCUSSION

We have recently shown that Itk‐deficient T cells have the ability to clear tumour cells without inducing GVHD, promoted by higher expression of Eomes and other molecules responsible for the cytotoxic effect against tumours.^22^ It is also known that Itk deficiency can result in the expansion of canonical Tregs.^14^, ^23^ Huang et al. showed that Tregs sorted from *Itk ^–/–^* mice did not ameliorate colitis in a model in *Rag^–/–^* mice. However, Gomez‐Rodriguez et al. reported that in vitro induced *Itk^–/–^* Tregs control colitis better than induced WT Tregs. Therefore, it remained unclear whether loss of Itk was beneficial in T cell‐driven disease. In this report, we examined the role of Tregs from *Itk^–/–^* mice in a GVHD model. Our data are in contrast with Huang et al, since we found that loss of Itk is beneficial in Tregs in the GVHD context. Our findings are novel as we showed that the loss of Itk gives rise not only to canTregs but also to ncTregs, and we showed that both canTregs and ncTregs from *Itk^–/–^* mice are functionally suppressive. We have also used a more clinically relevant GVHD and GVL model, and provided evidence that ncTregs from Itk‐deficient mice suppress GVHD, but had no effect on GVL function. This novel finding suggests that modulating ITK signaling may reduce T cell‐mediated GVHD without impacting GVL function.

Our data provide evidence that the loss of ITK does not result in aberrant FOXP3 expression in all T cell types, but rather that targeting ITK signaling gives rise to potent suppressive Tregs, and this intervention may have potential for T cell‐based therapeutics. We provided evidence that ncTregs are not the result of temporary activation, but that these cells are present post‐transplantation, suggesting that these cells are stable and could be used as a therapy.

Our data showed that the absence of Itk led to significantly higher percentages of canonical CD4^+^ CD25^+^FOXP3^+^ T cells (canTregs) and CD4^+^ CD25^–^FOXP3^+^ cells (ncTregs). Our data also showed that a higher proportion of these ncTregs express CTLA‐4, and carry a unique effector phenotype. This effector phenotype has been reported to be associated with more potent suppressive Tregs.^28^

Our data showed that CD8^+^ T cells from *Itk^–/–^* mice have a significantly higher frequency of central memory phenotype cells (CM), but CD4^+^ T cells from *Itk^–/–^* mice showed significantly increased expression of the effector memory (EM) phenotype, similar to what has previously been reported.^18^, ^19^, ^20^ Published data from both animal models and humans have demonstrated that effector memory T cells (T_EM_) and central memory T cells (T_CM_) from unprimed donors have a decreased ability to induce GVHD.^29^, ^30^ These data suggest that targeting ITK signaling has therapeutic potential. Our data also showed that a higher proportion of ncTregs from mice lacking Itk express CXCR3 than canTreg from WT mice. CXCR3^+^ Tregs are thought to be the Tregs with the most potent immune regulatory properties,^31^ suggesting that *Itk* ^–/−^ ncTregs have high suppressive potential. It has also been previously shown that CXCR3‐expressing Tregs can suppress the Th1 response.^32^ This would suggest that they can migrate and maintain their suppressive ability in the GVHD target organs longer than canonical Tregs from WT mice.^21^ We also confirmed that the canTregs and ncTregs from *Itk* ^–/−^ mice are maintaining their phenotype.

Our data showed that a higher proportion of ncTregs from *Itk^–/–^* mice express PD‐1, supporting published work that PD‐1 expression is critical for Treg function.^33^, ^34^ We also showed that a higher proportion of ncTregs from *Itk^–/–^* mice express ICOS. Published data have shown that ICOS‐deficient Tregs cannot suppress inflammation.^35^ Furthermore, there is evidence that IL‐2 therapy significantly affects ICOS‐expressing Tregs.^36^ Our data provided evidence that inhibition of ITK increases ncTregs which express ICOS, and high expression of ICOS is pivotal for Treg function.^37^

Published studies have shown that chemokine CCR7 is expressed on both naïve and effector/memory Tregs. Naïve Tregs lacking CCR7 in guts cannot recirculate, and published data have also shown that effector/memory Tregs lacking CCR7 expression accumulate in the site of inflammation.^38^, ^39^ Our data showed that the ncTregs express significantly less CCR7, suggesting that these Tregs may accumulate in sites of inflammatory GVHD damage. All of these observations were also confirmed with RNA sequencing analysis of ncTregs and canTregs.

We provided evidence that a higher proportion of ncTregs from Itk‐deficient mice express CTLA‐4, but no changes in IL‐10 (compared to WT) upon stimulation. Our data also showed that transplanting Tregs from *Itk* ^–/−^ mice led to a decrease in proinflammatory cytokines in the serum and less tissue damage in GVHD target organs induced by donor conventional T cells. Furthermore, treating mouse and human T cells with SLP76pTYR, a recently developed peptide inhibitor that specifically inhibits Itk kinase phosphorylation,^13^ led to an increase in in proportion expressing Foxp3 in vitro.

These results revealed that canonical Tregs from Itk‐deficient mice may have superior suppressive abilities compared to WT canonical Tregs. Due to technical and biological issues with sorting, we were not able to compare WT ncTregs with *Itk* ^–/−^ ncTregs in the in vivo experiments in order to see whether the effects are coming from the altered Itk signaling or from the noncanonical phenotype. However, we could compare the canonical Treg groups from *Itk* ^–/−^ and WT mice, as well as canTregs and ncTregs from *Itk* ^–/−^ mice, and observed that both Itk signaling and the noncanonical phenotype contribute separately to the differences in transcriptomic profiles.

Treg cells are usually administered in similar proportions to donor conventional T cells, and previous data suggested that a lower ratio of Treg cells, similar to their physiological proportion, has no protective effect against GVHD.^40^ In our experiments, we confirmed these findings. When we transplanted 1 million CD8^+^ T cells from WT mice and treated with 0.5 million CD25^+^FOXP3^+^ Tregs from WT mice, recipient mice were not protected from developing GVHD, although they cleared tumour cells. They also showed decreased serum cytokines and less tissue damage in small intestines on day 7. However, mice that were transplanted with WT canTregs started showing significant GVHD signs after day 10. In contrast, recipient mice transplanted with 1 million WT T cells and 0.5 million canonical *Itk* ^–/−^ Tregs had significantly delayed acute GVHD and increased survival rates compared to non‐treated and WT canTreg‐treated groups. Furthermore, we observed that recipient mice transplanted with 1 million WT T cells and 0.5 million *Itk* ^–/−^ ncTregs showed almost no signs of GVHD, and had increased survival rates compared to non‐treated and WT canTreg‐treated groups. This occurred despite the 1:2 Treg:Tconv ratio, suggesting that *Itk* ^–/−^ ncTregs may be more suppressive than canonical Tregs.

Tregs are critical for the maintenance of tolerance, and transfer of Treg cells can ameliorate GVHD by restoring defective tolerance mechanisms.^41^ One of the biggest challenges is to isolate functional Tregs in large numbers for immunotherapy.^42^ This challenge is due to the low number of Tregs in peripheral blood.^4^ Given their low numbers in the blood, it is difficult to obtain enough Tregs in the clinic for use as a therapy. If the number of collected canTregs and ncTregs could be increased for use in GVHD patients, such patients could be better protected from GVHD and other autoimmune disorders. This limitation in cell numbers could be mitigated by in vitro expansion of Tregs, but data have shown that in vitro expansion causes loss of FOXP3 expression.^11^ Other reports suggested that in vitro expansion of Tregs with rapamycin increases Tregs, but reduces suppressive function.^43^ Several recent protocols have been developed using Chimeric Antigen Receptor T‐Cell therapy. This is a very attractive approach, but this approach also has considerable limitations, such as problems with trafficking, downregulating chemokine receptors and cryopreservation.^45^, ^52^ Our RNA sequences data support our hypothesis that *Itk^–/–^* ncTregs have a more suppressive effector‐like and Tfr‐like phenotype. They also suggest that Itk could be a potential therapeutic target for increasing regulatory T cells that can ameliorate GVHD, and could be used in other autoimmune diseases.

Altogether, these data support our hypothesis that *Itk^–/–^* canTregs and ncTregs differentially regulate a number of important genes that are involved in immune responses to pathogens and alloantigens, and they also express more effector, cell adhesion and migration molecules. Even though we did not see such drastic genetic differences when we compared the *Itk^–/–^* canTregs and WT canTregs (versus in the comparison between ncTregs and canTregs), the functional data along with the DEGS and functional enrichment analysis suggest that the differences are not only coming from the noncanonical phenotype, but that Itk signaling also contributes to these effects.

The pharmacological treatments available to maintain FOXP3 expression, such as CPG methylation, histone deacetylase inhibitors and the mammalian target of rapamycin (mTOR) inhibitor rapamycin (sirolimus),^46^ can result in general immunosuppression, which could lead to tumour relapse and infection. Our data showed that mouse, healthy human and GVHD patient T cells treated with our specific peptide SLP76pTYR had increased FOXP3 expression (and canonical and noncanonical Tregs), suggesting that Itk/SLP76 inhibition could be an alternative approach.

The presence of cytokine storm is considered one of the hallmarks of GVHD pathogenesis,^47^ and studies in animal GVHD models have shown that the loss of Tregs during development leads to increased production of proinflammatory cytokines by allo‐reactive donor T cells.^48^ Our data also show that mouse, healthy human, and GVHD patient T cells treated with our specific peptide SLP76pTYR had reduced expression of proinflammatory cytokines. Modulation of cytokine production by donor T cells can exert direct effects on GVHD target tissues. This peptide also expands Tregs, and these Tregs provide a significant reduction in WT T cell‐mediated GVHD, suggesting that treatment of donor T cells with the SLP76pTYR peptide may be a way to modulate cytokine storm following allotransplant.

In summary, we have shown that genetic or pharmacological ablation of Itk in T cells leads to an expansion of canonical and noncanonical functional Tregs. Treatment with these Tregs, especially those which are noncanonical, leads to attenuation of GVHD with preservation of GVL effects, suggesting that inhibiting Itk may be a potential therapeutic target for treatment of GVHD and other diseases where Tregs are therapeutically useful.

## CONFLICT OF INTEREST

AA receives research support from 3M Corporation. The other authors declare no conflicts of interest.

## Supporting information


**Supplementary Figure 1. Quantified FOXP3 and CD25 expression in CD8^+^ and CD4^+^ T cells, related to Figure 2**. (A) Quantification of FOXP3 and CD25 expression in CD4^+^ T cells from naive WT or *Itk* ^–/−^ mice. (B,C) Quantification of FOXP3 and CD25 expression in CD8^+^ T cells from naive WT or *Itk* ^–/−^ mice. NS, *p* > .05; * *p* ≤ .05; ** *p* ≤ .01; *** *p* ≤ .001; **** *p* ≤ .0001 (*n* = 3 mice per group). Data were analyzed using one‐way ANOVA with Tukey's test, for statistics data from three to five independent experiments pooled.

**Supplementary Figure 2**. (A) MACS purified CD4^+^ T cells from WT and *Itk^–/–^* were gated on CD4, vs SSC‐A, and then CD25 and FOXP3. (B) Post‐sort donor cells were gated on CD4 vs SSC‐A to plot CD25 vs FOXP3. (C) Using flow cytometric analysis post transplanted donor T cells from WT mice spleen were gated CD3 vs H‐2K^b^, followed by CD4 vs CD8 gating, and then CD4 T cells were gated on CD25 vs FOXP3 markers. (D)At day 7 post‐transplant splenic donor Tregs from recipient mice that were transplanted with donor Tregs from *Itk^–/–^* mice were stained and gated for CD3, H‐2K^b^ markers to determine donor T cells, followed by CD4, CD8 gating. Then donor CD4^+^ T cells were gated on CD25 vs FOXP3 (**E**) Post transplanted donor Tregs from WT mice liver stained and plotted on CD3 vs H‐2K^b^ to identify donor T cells. These T cells were further gated on CD4 vs CD8, and CD4 T cells were to plotted for CD25 vs FOXP3. (F) Similar gating strategy were applied to donor cells from recipient BALB/c liver transplanted with donor Tregs from *Itk^–/–^* mice. Cells were gated on CD3, H‐2K^b^ to identify donor T cells, followed by CD4 vs CD8 gating, and CD4 T cells plotted for CD25 and FOXP3. (G) Quantitative analysis from donor Tregs from Figure 2C,D. NS, *p* > .05; * *p* ≤ .05; ** *p* ≤ .01; *** *p* ≤ .001; **** *p* ≤ .0001(*n* = 4 mice per group). Data were analyzed using one‐way ANOVA with Tukey's test.

**Supplementary Figure 3. Treg subsets and marker expression, related to Figure 2**. WT CD4^+^ T cells were obtained from WT C57Bl/6 mice and *Itk ^–/–^* mice, and either stained immediately or cultured for 6 h with or without anti‐CD3/anti‐CD28, in the presence of GolgiPlug. Cells were then stained for IL‐2, IL‐10, CD3, CD4, CD25 and FOXP3. (A) Quantification IL‐2 expression in cultured canonical Tregs (canTregs) from WT C57Bl/6 mice or *Itk^–/–^* mice with and without anti‐CD3/anti‐CD28 stimulation. (B) Quantification of IL‐2 expression in cultured noncanonical Tregs (ncTregs) from WT C57Bl/6 mice or *Itk^–/–^* mice with and without anti‐CD3/anti‐CD28 stimulation. (C) Quantification of IL‐10 expression in cultured canTregs from WT C57Bl/6 mice or *Itk^–/–^* mice with and without anti‐CD3/anti‐CD28 stimulation. (D) Quantification of IL‐10 expression in cultured ncTregs from WT C57Bl/6 mice or *Itk^–/–^* mice with and without anti‐CD3/anti‐CD28 stimulation. NS, *p* > .05; * *p* ≤ .05; ** *p* ≤ .01; *** *p* ≤ .001; **** *p* ≤ .0001(*n* = 3 mice per group, 3 combined experiments are shown). Data were analyzed using one‐way ANOVA with Tukey's test, for statistics data from three independent experiments pooled.

**Supplementary Figure 4. Treg subset phenotypic changes, related to Figure 2**. (A,B) Canonical Tregs and noncanonical Tregs from naive WT C57Bl/6 mice and *Itk ^–/–^* mice were examined for CD44 and CD62L expression. (A) Quantification of effector Treg phenotype and (B) quantification of naive Treg phenotype for canonical Tregs and noncanonical Tregs from WT mice and *Itk ^–/–^* mice, based on CD44 and CD62L expression. (C,D) Quantification of memory phenotype (also using CD44 and CD62L expression) in conventional (C) CD4^+^and (D) CD8^+^ T cells from naive WT C57Bl/6 mice and *Itk ^–/–^* mice. NS, *p* > .05; * *p* ≤ .05; ** *p* ≤ .01; *** *p* ≤ .001; **** *p* ≤ .0001(*n* = 2 or 3 mice per group, 3 combined experiments are shown). Data were analyzed using one‐way ANOVA with Tukey's test, for statistics data from two to three independent experiments pooled.

**Supplementary Figure 5. Treg subset changes in CXCR3, PD‐1, ICOS, and CCR7 expression, related to Figure 2. (**A) Quantification of CXCR3 expression on canonical and noncanonical Tregs from naive WT C57Bl/6 mice and *Itk ^–/–^* mice. (B) Quantification of PD‐1 expression on canonical and noncanonical Tregs from naive WT C57Bl/6 mice and *Itk ^–/–^* mice. (C) Quantification of ICOS expression on canonical and noncanonical Tregs from naive WT C57Bl/6 mice and *Itk ^–/–^* mice. (D) Quantification of CCR7 expression on canonical and noncanonical Tregs from naive WT C57Bl/6 mice and *Itk ^–/–^* mice. NS, *p* > .05; * *p* ≤ .05; ** *p* ≤ .01; *** *p* ≤ .001; **** *p* ≤ .0001(*n* = 3 mice per group, one experiment is shown). Data were analyzed using one‐way ANOVA with Tukey's test, for statistics data from two to three independent experiments pooled.

**Supplementary Figure 6. Treatment with Tregs *in vivo* results in less damage to GVHD target organs, related to Figure 5**. BALB/c recipient mice were lethally irradiated and transplanted with 10 × 10^6^ T cell‐depleted bone marrow cells and 1 × 10^6^ WT‐*luc* ^+^ CD8^+^ T cells (donor T cells expressing luciferase). *Group 1* recipient mice were not given any additional cells (non‐treated). *Group 2* BALB/c recipient mice were treated with FACS sorted canonical Tregs from WT C57Bl/6 mice. *Group 3* BALB/c recipient mice were treated with FACS sorted canonical Tregs from *Itk* ^–/−^ mice. *Group 4* BALB/c recipient mice were treated with FACS sorted noncanonical Tregs from *Itk* ^–/−^ mice. At 7 days post‐transplant, livers and small intestines were taken from recipient mice, sectioned, stained with H&E, and photographed (see Fig. 6). Tissues obtained from recipient mice were graded for GVHD. Quantified GVHD scores for different groups are shown. Kruskal Wallis test was used for statistical analysis of GVHD grades. Dunn's multiple comparison test performed to determine statistical difference between groups. NS, *p *> .05; * *p* ≤ .05; ** *p* ≤ .01; *** *p* ≤ .001; **** *p* ≤ .0001 (*n* = 3 mice per group, one experiment shown).

**Supplementary Figure 7. Tregs from mouse, healthy human, and GVHD patients’ samples following SLP76pTYR treatment, related to Figure 8**. (A) Quantification of healthy human total Tregs in cells treated with SLP76pTYR or vehicle alone. (B) Quantification of healthy human noncanonical Tregs in cells treated with SLP76pTYR or vehicle alone. (C) Quantification of GVHD patient noncanonical Tregs in cells treated with SLP76pTYR or vehicle alone. (D) Quantification of mouse canTregs cells treated with SLP76pTYR or vehicle alone for 5 to 24 h. (E) Quantification of GVHD patient canonical Tregs in cells treated with SLP76pTYR or vehicle alone. (F) Quantification of healthy human canonical Tregs in cells treated with SLP76pTYR or vehicle alone. NS, *p* > .05; * *p* ≤ .05; ** *p* ≤ .01; *** *p* ≤ .001; **** *p* ≤ .0001. *n* = 3 per group for A, B, D, F; *n *= 5 per group for C, E; combined data from three independent experiments is shown. Data were analyzed using *t‐*test.

**Supplementary Figure 8. Disruption of Itk/SLP76 Y145 signaling decreased proinflammatory cytokines in healthy human and GVHD patient samples, related to Figure 8**. Human GVHD samples were treated with vehicle or SLP76pTYR, and cultured in the presence of GolgiPlug for 6 h. (A) IFN‐γ and TNF‐α expression of CD8^+^ T cells in culture. (B) IFN‐γ and TNF‐α expression of CD4^+^ T cells in culture. Cultured cells were not stimulated with anti‐CD3/anti‐CD28 (as in Fig. 8), but were treated with vehicle alone or SLP76pTYR.

**Supplementary Figure 9. SLP76pTYR peptide does not induce apoptosis in healthy human T cells, related to Figure 8**. Primary human PBMCs from healthy human donors were cultured for 5 h with and without SLP76pTYR, and were stained for CD3, CD4, Annexin V, and LIVE/DEAD Near‐IR. (A) Flow cytometry plots of human CD4^+^ T cells stained for Annexin V and Near‐IR. (B) Quantification of (A). (C) Flow cytometry plots of human CD8^+^ T cells stained for Annexin V and Near‐IR. (D) Quantification of (C). Statistical analysis was performed using one‐way ANOVA with Tukey's test. NS, *p* > .05; *p ≤ .05; **p ≤ .01; ***p ≤ .001; ****p ≤ .0001. (*n* = 5 mice per group, one experiment shown).

**Supplementary Figure 10. SLP76pTYR peptide is not toxic to mouse T cells and does not induce apoptosis in mouse cells, related to Figure 8**. WT mouse CD4^+^ and CD8^+^ T cells were cultured for 5 h in the presence of SLP76pTYR or vehicle alone, and were stained for CD3, CD4, Annexin V, and Near‐IR. (A) Flow cytometry plots of mouse CD4^+^ T cells for Annexin and Near‐IR. (B) Quantification of several samples of CD4^+^ T cells for live, apoptotic, and dead cells. (C) Flow cytometry plots of mouse CD8^+^ T cells for Annexin and Near‐IR. (D) Quantification of several samples of CD8^+^ T cells for live, apoptotic, and dead cells. (E‐H) Mouse T cell lymphoma (Yac‐1) cells were transduced with GFP and luciferase and were cultured in the presence of SLP76pTYR or vehicle alone. Bioluminescence of Yac‐1 cells was quantified by adding luciferin to the cells at (E) 0 h, (F) 2 h, (G) 4 h, and (H) 6 h, and imaging them with the IVIS 50 system. Statistical analysis was performed by using two‐way ANOVA and Tukey's test. NS, *p* > .05; *p ≤ .05; **p ≤ .01; ***p ≤ .001; ****p ≤ .0001. (*n* = 3 mice per group, one experiment shown).
