# Supplementary figures and images for: Interleukin‐2‐inducible T‐cell kinase (Itk) signaling regulates potent noncanonical regulatory T cells
Source: Clin Transl Med. 2021 Dec 17;11(12):e625. doi: 10.1002/ctm2.625 (PMC8679839; doi:10.1002/ctm2.625)

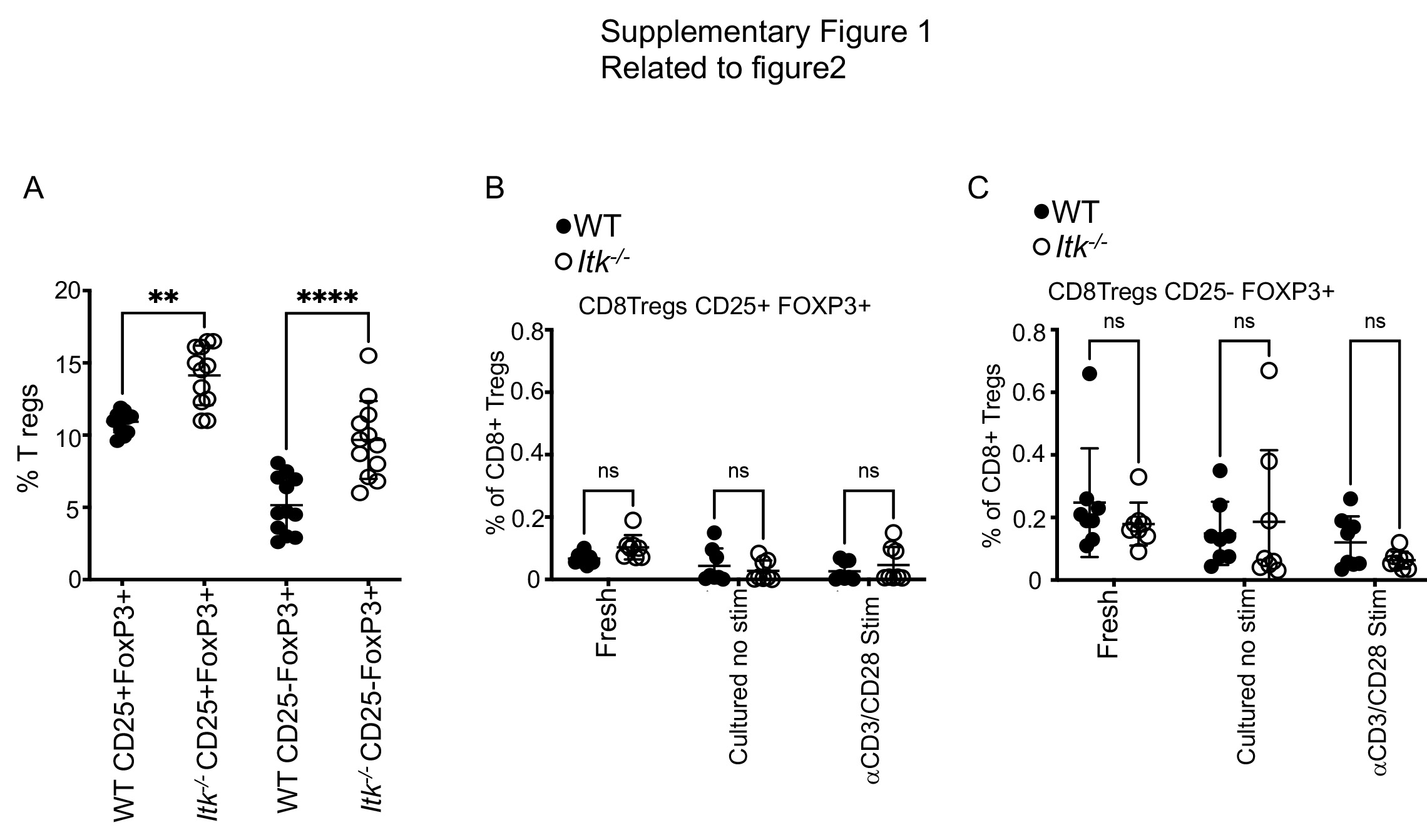

Supplement: Supplementary file 1 — Supplementary Figure 1. Quantified FOXP3 and CD25 expression in CD8+ and CD4+ T cells, related to Figure 2. (A) Quantification of FOXP3 and CD25 expression in CD4+ T cells from naive WT or Itk –/− mice. (B,C) Quantification of FOXP3 and CD25 expression in CD8+ T cells from naive WT or Itk –/− mice. NS, p > .05; * p ≤ .05; ** p ≤ .01; *** p ≤ .001; **** p ≤ .0001 (n = 3 mice per group). Data were analyzed using one‐way ANOVA with Tukey's test, for statistics data from three to five independent experiments pooled. [file CTM2-11-e625-s004.jpg]

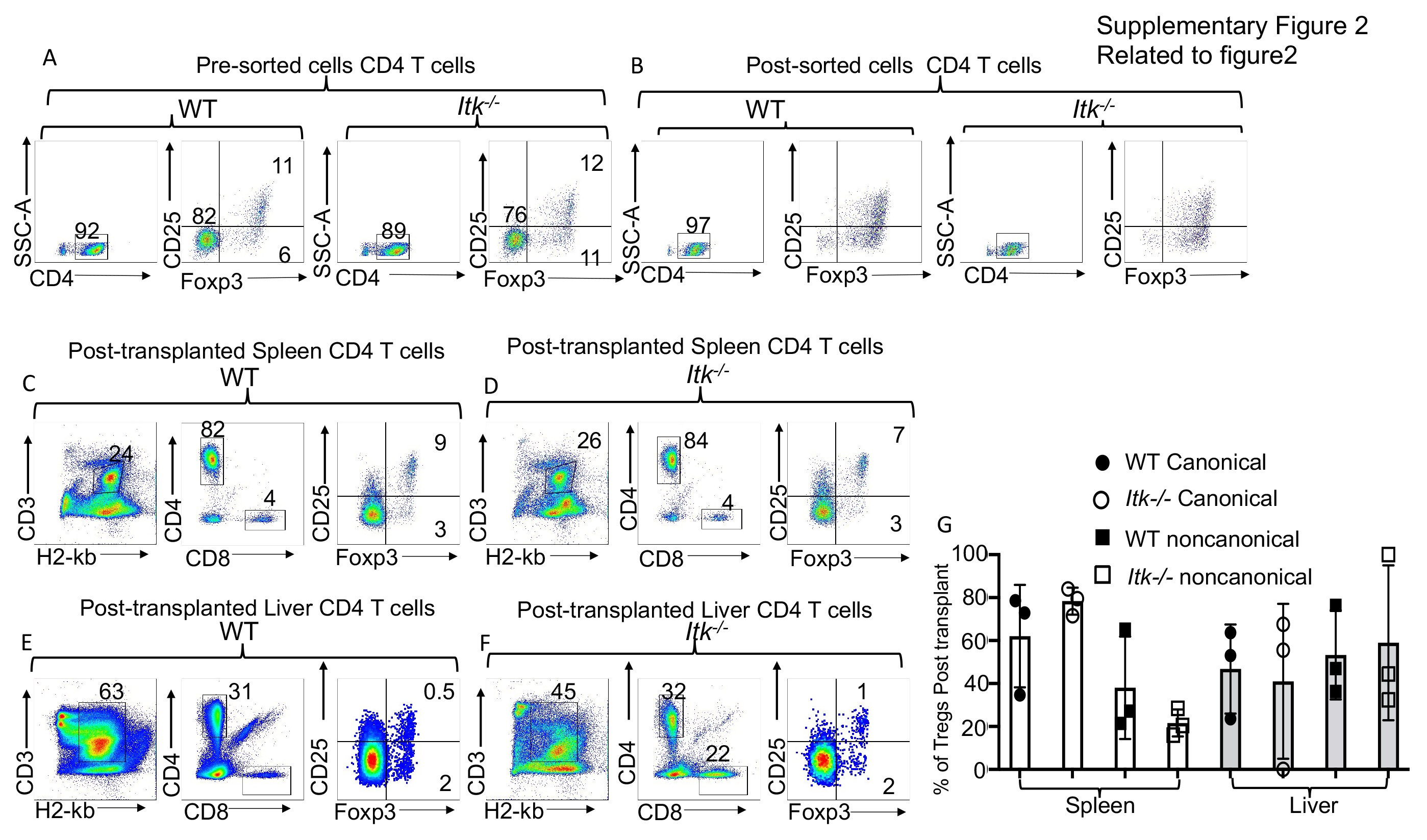

Supplement: Supplementary file 2 — Supplementary Figure 2. (A) MACS purified CD4+ T cells from WT and Itk–/– were gated on CD4, vs SSC‐A, and then CD25 and FOXP3. (B) Post‐sort donor cells were gated on CD4 vs SSC‐A to plot CD25 vs FOXP3. (C) Using flow cytometric analysis post transplanted donor T cells from WT mice spleen were gated CD3 vs H‐2Kb, followed by CD4 vs CD8 gating, and then CD4 T cells were gated on CD25 vs FOXP3 markers. (D)At day 7 post‐transplant splenic donor Tregs from recipient mice that were transplanted with donor Tregs from Itk–/– mice were stained and gated for CD3, H‐2Kb markers to determine donor T cells, followed by CD4, CD8 gating. Then donor CD4+ T cells were gated on CD25 vs FOXP3 (E) Post transplanted donor Tregs from WT mice liver stained and plotted on CD3 vs H‐2Kb to identify donor T cells. These T cells were further gated on CD4 vs CD8, and CD4 T cells were to plotted for CD25 vs FOXP3. (F) Similar gating strategy were applied to donor cells from recipient BALB/c liver transplanted with donor Tregs from Itk–/– mice. Cells were gated on CD3, H‐2Kb to identify donor T cells, followed by CD4 vs CD8 gating, and CD4 T cells plotted for CD25 and FOXP3. (G) Quantitative analysis from donor Tregs from Figure 2C,D. NS, p > .05; * p ≤ .05; ** p ≤ .01; *** p ≤ .001; **** p ≤ .0001(n = 4 mice per group). Data were analyzed using one‐way ANOVA with Tukey's test. [file CTM2-11-e625-s008.jpg]

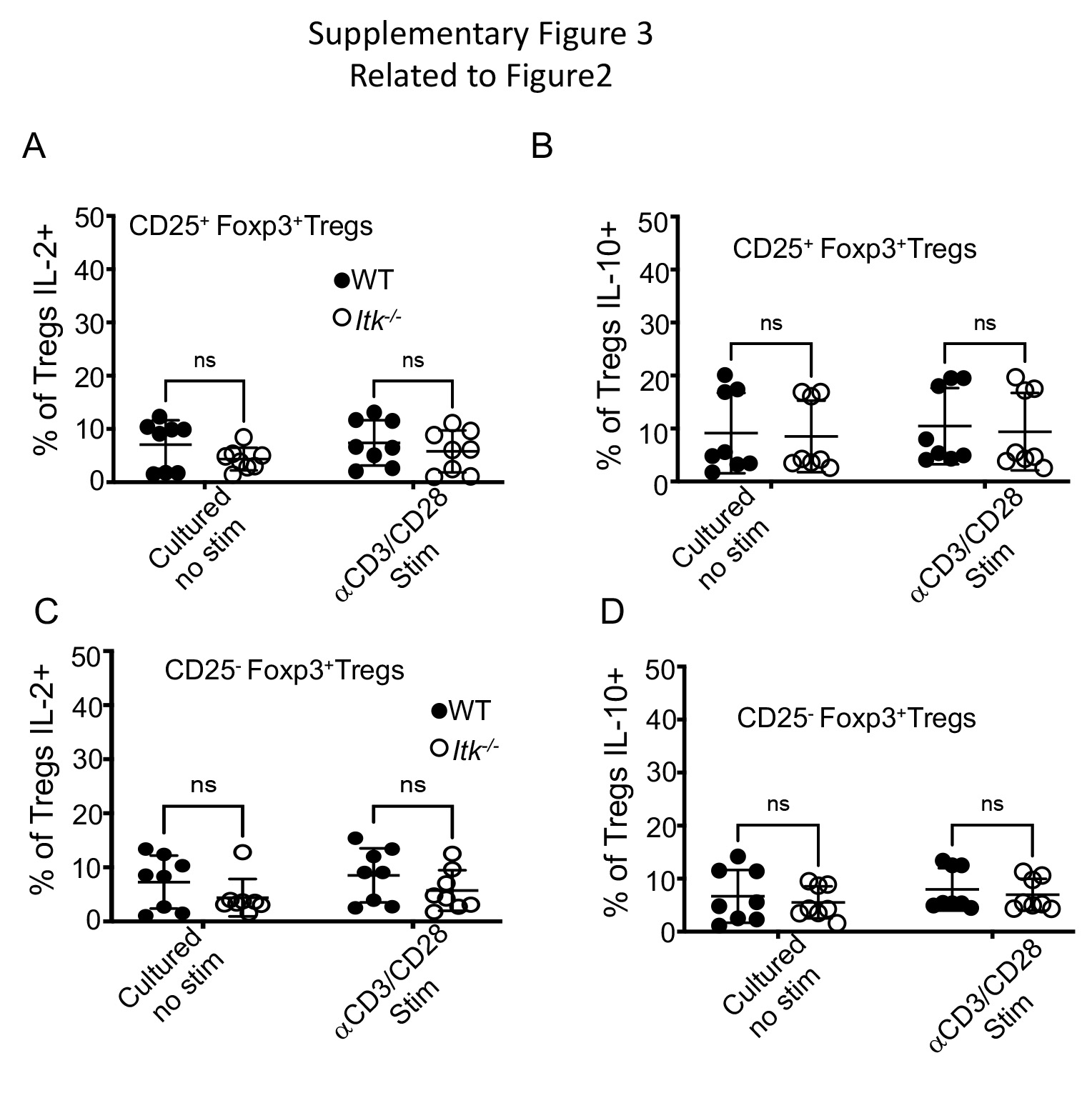

Supplement: Supplementary file 3 — Supplementary Figure 3. Treg subsets and marker expression, related to Figure 2. WT CD4+ T cells were obtained from WT C57Bl/6 mice and Itk –/– mice, and either stained immediately or cultured for 6 h with or without anti‐CD3/anti‐CD28, in the presence of GolgiPlug. Cells were then stained for IL‐2, IL‐10, CD3, CD4, CD25 and FOXP3. (A) Quantification IL‐2 expression in cultured canonical Tregs (canTregs) from WT C57Bl/6 mice or Itk–/– mice with and without anti‐CD3/anti‐CD28 stimulation. (B) Quantification of IL‐2 expression in cultured noncanonical Tregs (ncTregs) from WT C57Bl/6 mice or Itk–/– mice with and without anti‐CD3/anti‐CD28 stimulation. (C) Quantification of IL‐10 expression in cultured canTregs from WT C57Bl/6 mice or Itk–/– mice with and without anti‐CD3/anti‐CD28 stimulation. (D) Quantification of IL‐10 expression in cultured ncTregs from WT C57Bl/6 mice or Itk–/– mice with and without anti‐CD3/anti‐CD28 stimulation. NS, p > .05; * p ≤ .05; ** p ≤ .01; *** p ≤ .001; **** p ≤ .0001(n = 3 mice per group, 3 combined experiments are shown). Data were analyzed using one‐way ANOVA with Tukey's test, for statistics data from three independent experiments pooled. [file CTM2-11-e625-s001.jpg]

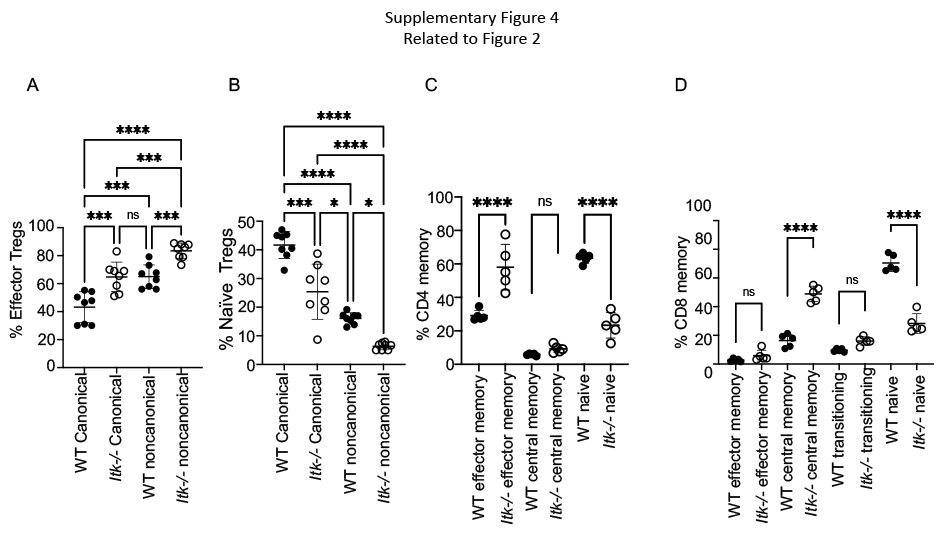

Supplement: Supplementary file 4 — Supplementary Figure 4. Treg subset phenotypic changes, related to Figure 2. (A,B) Canonical Tregs and noncanonical Tregs from naive WT C57Bl/6 mice and Itk –/– mice were examined for CD44 and CD62L expression. (A) Quantification of effector Treg phenotype and (B) quantification of naive Treg phenotype for canonical Tregs and noncanonical Tregs from WT mice and Itk –/– mice, based on CD44 and CD62L expression. (C,D) Quantification of memory phenotype (also using CD44 and CD62L expression) in conventional (C) CD4+and (D) CD8+ T cells from naive WT C57Bl/6 mice and Itk –/– mice. NS, p > .05; * p ≤ .05; ** p ≤ .01; *** p ≤ .001; **** p ≤ .0001(n = 2 or 3 mice per group, 3 combined experiments are shown). Data were analyzed using one‐way ANOVA with Tukey's test, for statistics data from two to three independent experiments pooled. [file CTM2-11-e625-s006.jpg]

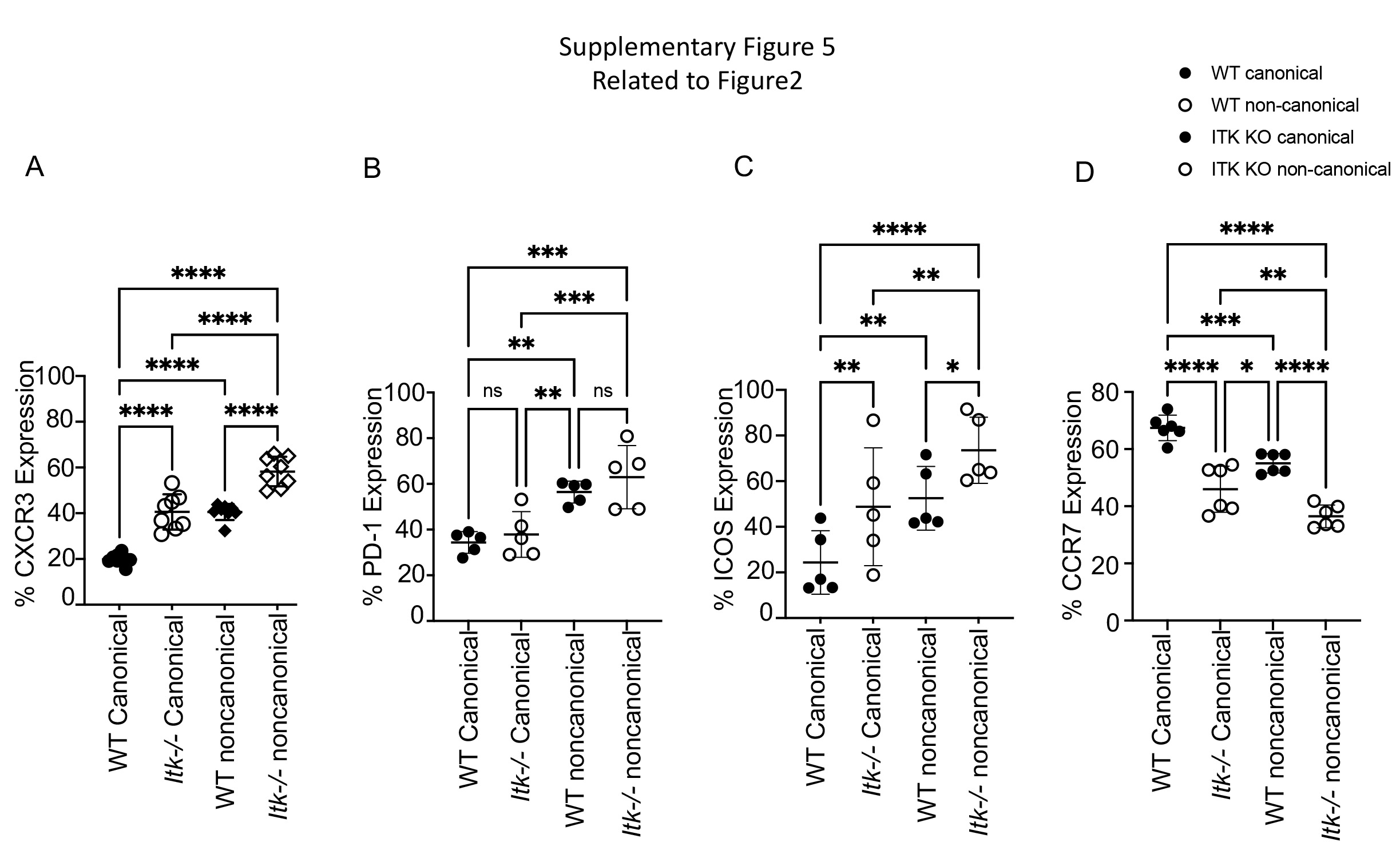

Supplement: Supplementary file 5 — Supplementary Figure 5. Treg subset changes in CXCR3, PD‐1, ICOS, and CCR7 expression, related to Figure 2. (A) Quantification of CXCR3 expression on canonical and noncanonical Tregs from naive WT C57Bl/6 mice and Itk –/– mice. (B) Quantification of PD‐1 expression on canonical and noncanonical Tregs from naive WT C57Bl/6 mice and Itk –/– mice. (C) Quantification of ICOS expression on canonical and noncanonical Tregs from naive WT C57Bl/6 mice and Itk –/– mice. (D) Quantification of CCR7 expression on canonical and noncanonical Tregs from naive WT C57Bl/6 mice and Itk –/– mice. NS, p > .05; * p ≤ .05; ** p ≤ .01; *** p ≤ .001; **** p ≤ .0001(n = 3 mice per group, one experiment is shown). Data were analyzed using one‐way ANOVA with Tukey's test, for statistics data from two to three independent experiments pooled. [file CTM2-11-e625-s007.jpg]

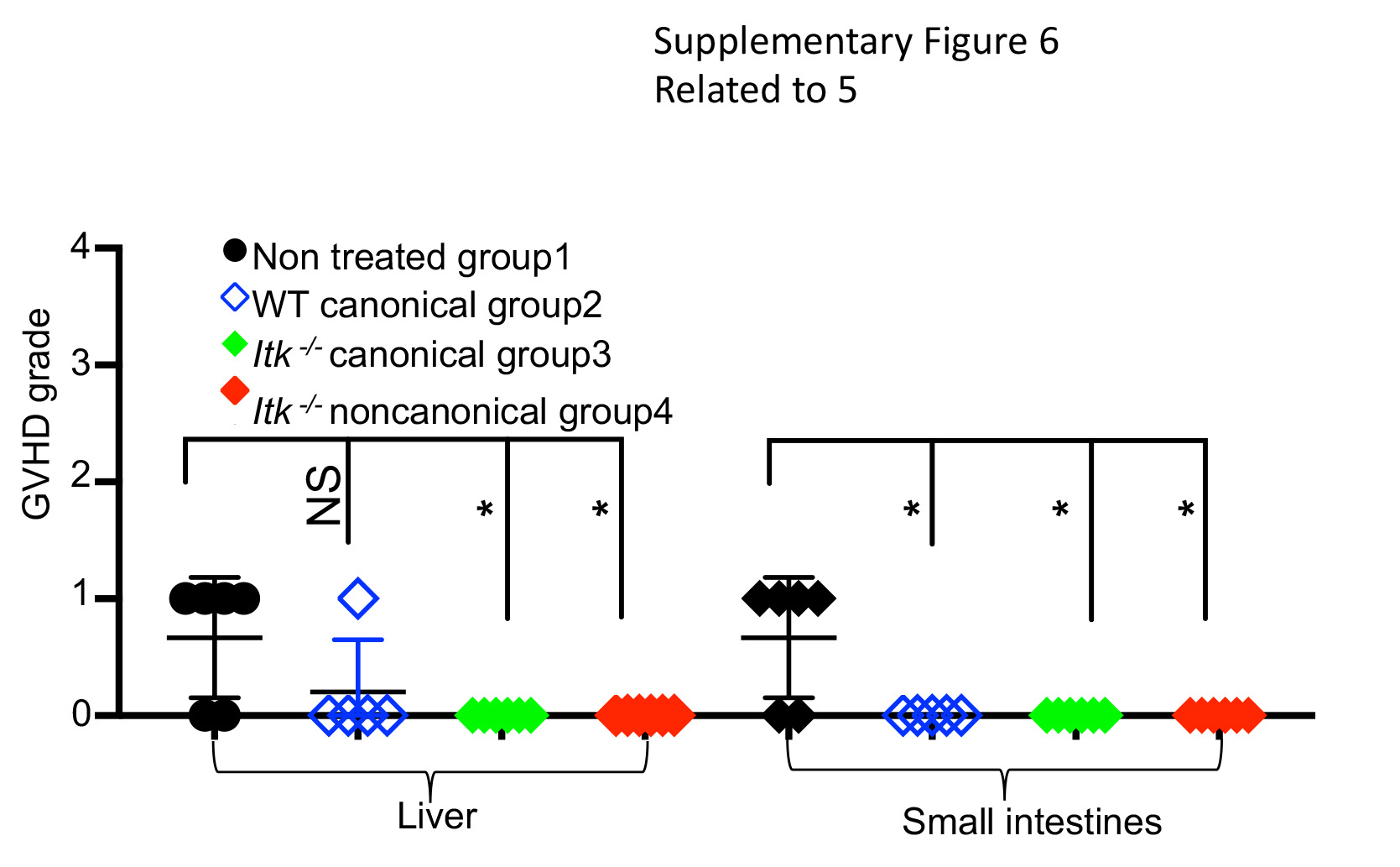

Supplement: Supplementary file 6 — Supplementary Figure 6. Treatment with Tregs in vivo results in less damage to GVHD target organs, related to Figure 5. BALB/c recipient mice were lethally irradiated and transplanted with 10 × 106 T cell‐depleted bone marrow cells and 1 × 10^6^ WT‐luc + CD8+ T cells (donor T cells expressing luciferase). Group 1 recipient mice were not given any additional cells (non‐treated). Group 2 BALB/c recipient mice were treated with FACS sorted canonical Tregs from WT C57Bl/6 mice. Group 3 BALB/c recipient mice were treated with FACS sorted canonical Tregs from Itk –/− mice. Group 4 BALB/c recipient mice were treated with FACS sorted noncanonical Tregs from Itk –/− mice. At 7 days post‐transplant, livers and small intestines were taken from recipient mice, sectioned, stained with H&E, and photographed (see Fig. 6). Tissues obtained from recipient mice were graded for GVHD. Quantified GVHD scores for different groups are shown. Kruskal Wallis test was used for statistical analysis of GVHD grades. Dunn's multiple comparison test performed to determine statistical difference between groups. NS, p > .05; * p ≤ .05; ** p ≤ .01; *** p ≤ .001; **** p ≤ .0001 (n = 3 mice per group, one experiment shown). [file CTM2-11-e625-s003.jpg]

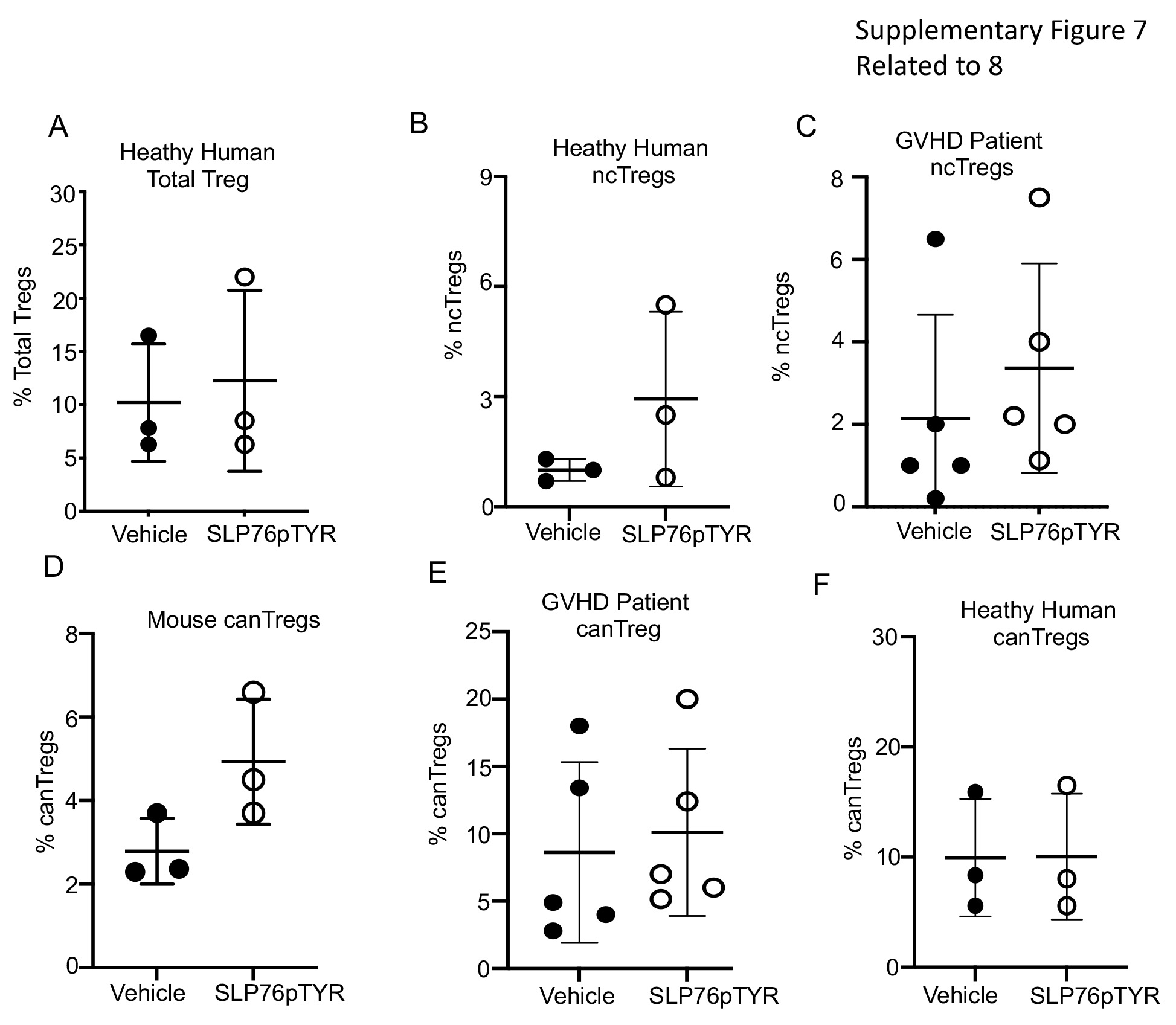

Supplement: Supplementary file 7 — Supplementary Figure 7. Tregs from mouse, healthy human, and GVHD patients’ samples following SLP76pTYR treatment, related to Figure 8. (A) Quantification of healthy human total Tregs in cells treated with SLP76pTYR or vehicle alone. (B) Quantification of healthy human noncanonical Tregs in cells treated with SLP76pTYR or vehicle alone. (C) Quantification of GVHD patient noncanonical Tregs in cells treated with SLP76pTYR or vehicle alone. (D) Quantification of mouse canTregs cells treated with SLP76pTYR or vehicle alone for 5 to 24 h. (E) Quantification of GVHD patient canonical Tregs in cells treated with SLP76pTYR or vehicle alone. (F) Quantification of healthy human canonical Tregs in cells treated with SLP76pTYR or vehicle alone. NS, p > .05; * p ≤ .05; ** p ≤ .01; *** p ≤ .001; **** p ≤ .0001. n = 3 per group for A, B, D, F; n = 5 per group for C, E; combined data from three independent experiments is shown. Data were analyzed using t‐test. [file CTM2-11-e625-s002.jpg]

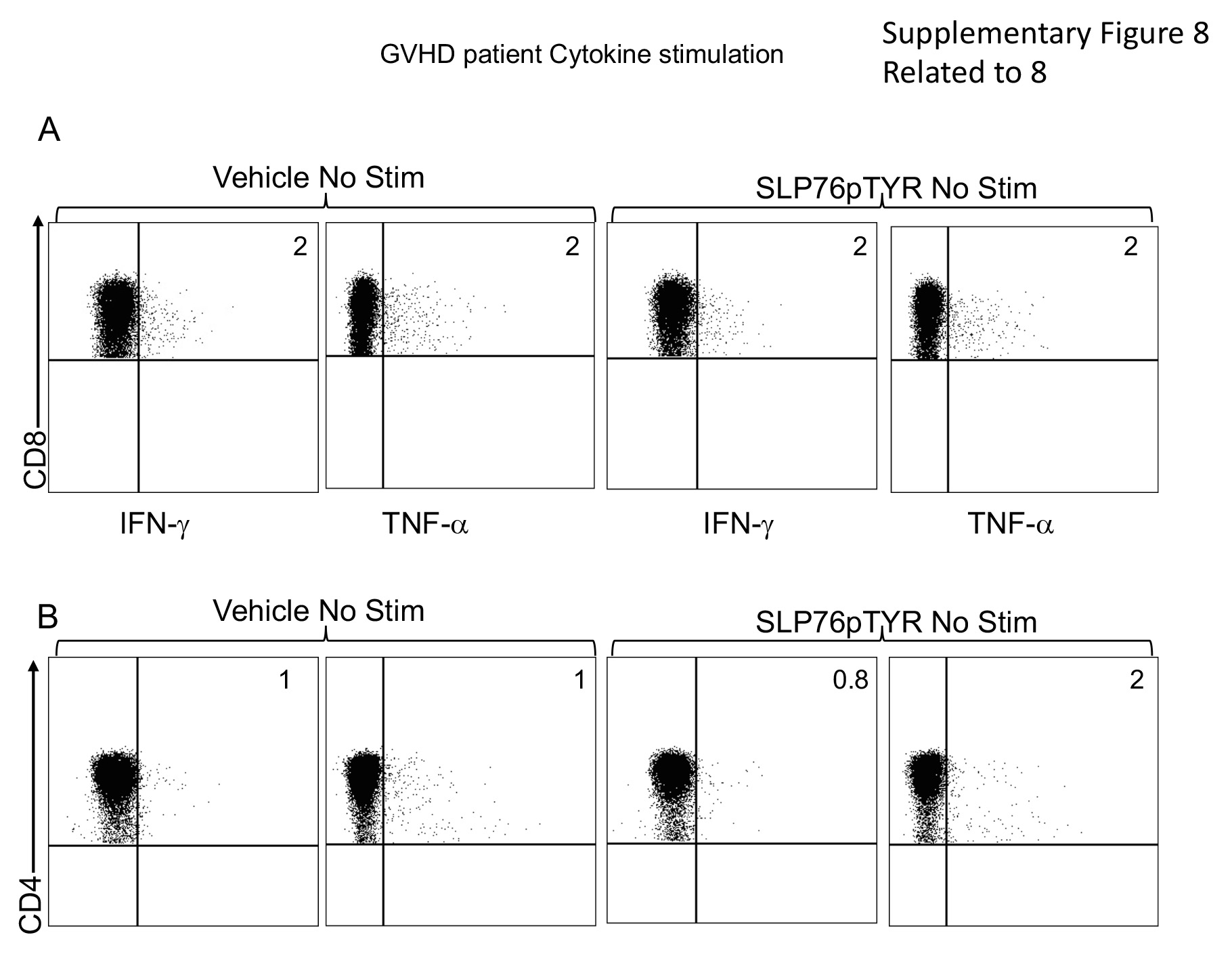

Supplement: Supplementary file 8 — Supplementary Figure 8. Disruption of Itk/SLP76 Y145 signaling decreased proinflammatory cytokines in healthy human and GVHD patient samples, related to Figure 8. Human GVHD samples were treated with vehicle or SLP76pTYR, and cultured in the presence of GolgiPlug for 6 h. (A) IFN‐γ and TNF‐α expression of CD8+ T cells in culture. (B) IFN‐γ and TNF‐α expression of CD4+ T cells in culture. Cultured cells were not stimulated with anti‐CD3/anti‐CD28 (as in Fig. 8), but were treated with vehicle alone or SLP76pTYR. [file CTM2-11-e625-s005.jpg]

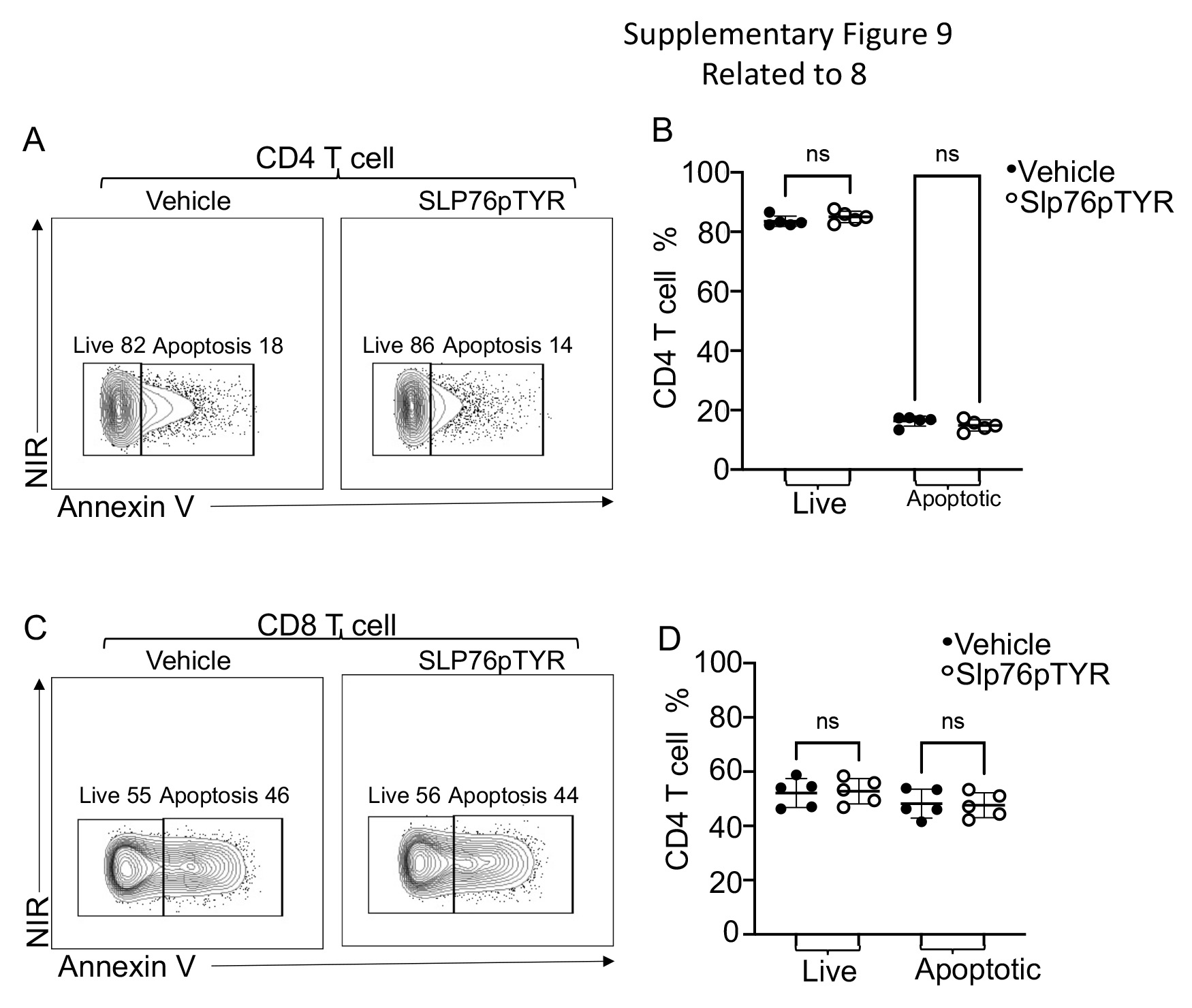

Supplement: Supplementary file 9 — Supplementary Figure 9. SLP76pTYR peptide does not induce apoptosis in healthy human T cells, related to Figure 8. Primary human PBMCs from healthy human donors were cultured for 5 h with and without SLP76pTYR, and were stained for CD3, CD4, Annexin V, and LIVE/DEAD Near‐IR. (A) Flow cytometry plots of human CD4+ T cells stained for Annexin V and Near‐IR. (B) Quantification of (A). (C) Flow cytometry plots of human CD8+ T cells stained for Annexin V and Near‐IR. (D) Quantification of (C). Statistical analysis was performed using one‐way ANOVA with Tukey's test. NS, p > .05; *p ≤ .05; **p ≤ .01; ***p ≤ .001; ****p ≤ .0001. (n = 5 mice per group, one experiment shown). [file CTM2-11-e625-s009.jpg]

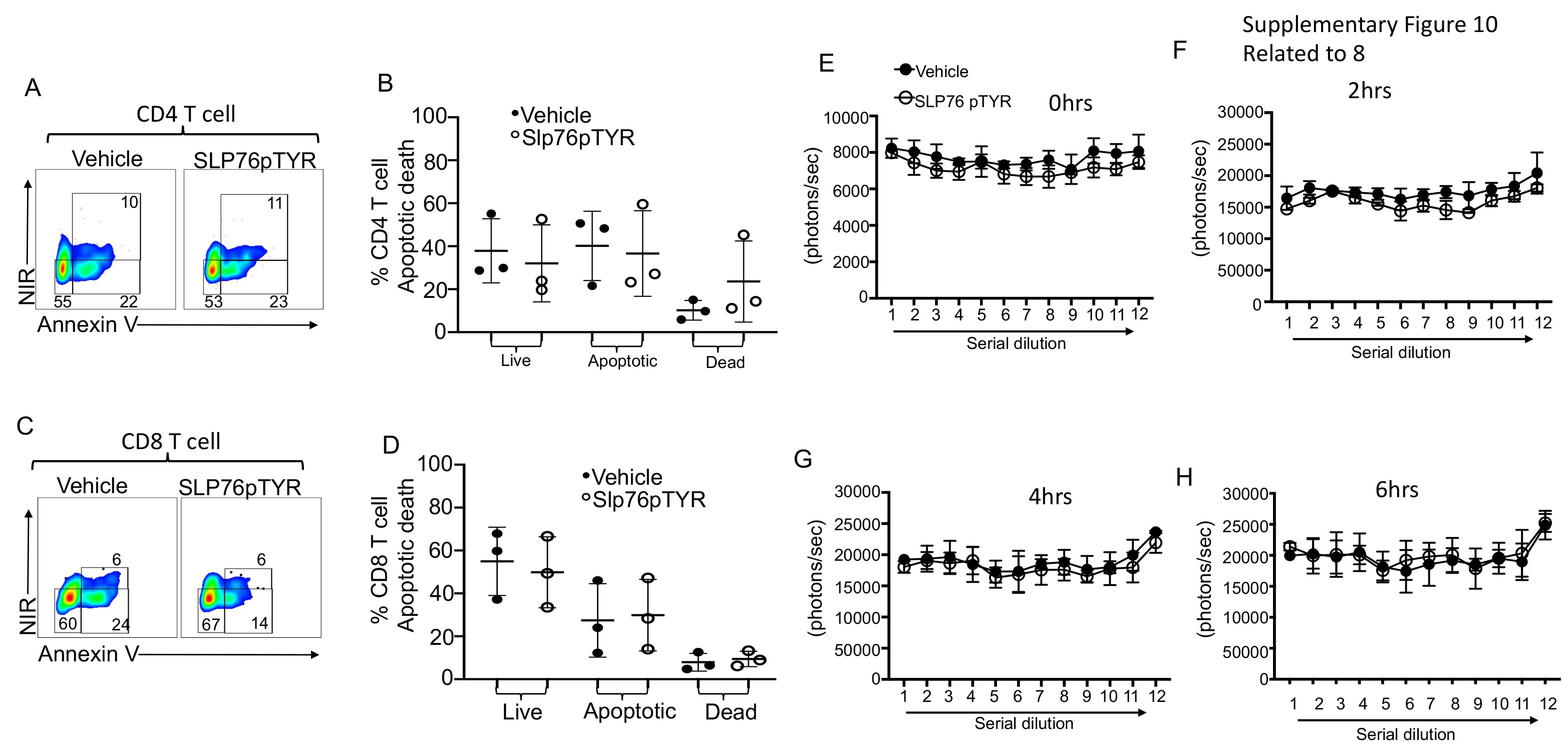

Supplement: Supplementary file 10 — Supplementary Figure 10. SLP76pTYR peptide is not toxic to mouse T cells and does not induce apoptosis in mouse cells, related to Figure 8. WT mouse CD4+ and CD8+ T cells were cultured for 5 h in the presence of SLP76pTYR or vehicle alone, and were stained for CD3, CD4, Annexin V, and Near‐IR. (A) Flow cytometry plots of mouse CD4+ T cells for Annexin and Near‐IR. (B) Quantification of several samples of CD4+ T cells for live, apoptotic, and dead cells. (C) Flow cytometry plots of mouse CD8+ T cells for Annexin and Near‐IR. (D) Quantification of several samples of CD8+ T cells for live, apoptotic, and dead cells. (E‐H) Mouse T cell lymphoma (Yac‐1) cells were transduced with GFP and luciferase and were cultured in the presence of SLP76pTYR or vehicle alone. Bioluminescence of Yac‐1 cells was quantified by adding luciferin to the cells at (E) 0 h, (F) 2 h, (G) 4 h, and (H) 6 h, and imaging them with the IVIS 50 system. Statistical analysis was performed by using two‐way ANOVA and Tukey's test. NS, p > .05; *p ≤ .05; **p ≤ .01; ***p ≤ .001; ****p ≤ .0001. (n = 3 mice per group, one experiment shown). [file CTM2-11-e625-s010.jpg]
